# Residue–Residue Contact Can Be a Potential Feature for the Prediction of Lysine Crotonylation Sites

**DOI:** 10.3389/fgene.2021.788467

**Published:** 2022-01-04

**Authors:** Rulan Wang, Zhuo Wang, Zhongyan Li, Tzong-Yi Lee

**Affiliations:** ^1^ School of Science and Engineering, The Chinese University of Hong Kong, Shenzhen, China; ^2^ Warshel Institute for Computational Biology, The Chinese University of Hong Kong, Shenzhen, China; ^3^ School of Life and Health Sciences, The Chinese University of Hong Kong, Shenzhen, China

**Keywords:** post-translation modification, crotonylation site prediction, residue–residue contact, machine learning, supervised learning

## Abstract

Lysine crotonylation (Kcr) is involved in plenty of activities in the human body. Various technologies have been developed for Kcr prediction. Sequence-based features are typically adopted in existing methods, in which only linearly neighboring amino acid composition was considered. However, modified Kcr sites are neighbored by not only the linear-neighboring amino acid but also those spatially surrounding residues around the target site. In this paper, we have used residue–residue contact as a new feature for Kcr prediction, in which features encoded with not only linearly surrounding residues but also those spatially nearby the target site. Then, the spatial-surrounding residue was used as a new scheme for feature encoding for the first time, named residue–residue composition (RRC) and residue–residue pair composition (RRPC), which were used in supervised learning classification for Kcr prediction. As the result suggests, RRC and RRPC have achieved the best performance of RRC at an accuracy of 0.77 and an area under curve (AUC) value of 0.78, RRPC at an accuracy of 0.74, and an AUC value of 0.80. In order to show that the spatial feature is of a competitively high significance as other sequence-based features, feature selection was carried on those sequence-based features together with feature RRPC. In addition, different ranges of the surrounding amino acid compositions’ radii were used for comparison of the performance. After result assessment, RRC and RRPC features have shown competitively outstanding performance as others or in some cases even around 0.20 higher in accuracy or 0.3 higher in AUC values compared with sequence-based features.

## 1 Introduction

Post-translational modifications (PTMs) have great impacts on regulating the activity of most eukaryote proteins Mann and Jensen (2003), which play significant roles in numerous biological processes by modulating regulation of protein functions and cellular processes Huang et al. (2017). For instance, histone acetylation plays a pivotal role in mammalian DNA repair Gong and Miller (2013). Sumoylation was found on transcription factors with greatly increased frequencies, which shows that it has a large impact on the transcription of protein Filtz et al. (2014), Huang et al. (2019). The ubiquitin–proteasome pathway is the most important protein degradation pathway in eukaryotic cells and participates in various physiological processes, including transcription regulation, the cell cycle, apoptosis, DNA damage repair, the metabolism, and immunity Tu et al. (2012), Wang et al. (2020), and ubiquitination is an important post-translational modification, which controls protein turnover and also serves intriguing non-proteolytic regulatory functions Li et al. (2021). Protein–protein interactions Li et al. (2012), Vermeulen et al. (2008), signaling pathways Hornbeck et al. (2015), apoptosis Zamaraev et al. (2017), cell death Urdinguio et al. (2019), cell regulation and pathogenesis Chung et al. (2020), and metabolic pathways Cruz et al. (2019), Romero-Puertas and Sandalio (2016) are all affected by various kinds of PTMs. Due to its importance, plenty of datasets with annotated PTMs of various types have been released in decades, such as emerging S-sulfenylation Bui et al. (2016), S-glutathionylation Chen et al. (2015), and succinylation Huang et al. (2015), Kao et al. (2020), which provided enough resources for investigation. Besides those earlier-discovered PTMs, crotonylation is a recently discovered one, which was originally found in somatic and mouse male germ cells and enriched on sex chromosomes Tan et al. (2011), and of paramount importance in regulating various biological processes. The abundance of MS-verified crotonylated peptides enabled the investigation of substrate site specificity of crotonylation sites through sequence-based attributes Huang et al. (2018). In 2017, Ju, Z. and He, J.J. had proposed an SVM-based method by using attribute CKSAAP for this prediction, and a tool named CKSAAP_CrotSite was developed at that time Ju and He (2017); also in 2017, Wang, R.Q. had proposed another method based on ensemble RF, which employed the attribute of pseudo-AAC Qiu et al. (2018). In 2018, 5,995 sites on 2,120 proteins had first been extracted and released by Liu, K. et al. Liu et al. (2018) and provided more experimental-verified crotonylated samples in plant Carica papaya L, which filled in the gaps of lacking samples in computational analysis of crotonylation. Based on these Carica papaya L. data, Zhao, Y et al. have carried out a prediction on the large dataset, in which the deep learning method has been involved Zhao et al. (2020) and more deep learning-related methods were released, such as “DeepKcr”Lv et al. (2021) and “nhKcr”Chen Y. Z. et al. (2021). These tools have highly improved the ablities of Kcr modification. However, these predictions are all based on sequence-based features, which consist of a piece of peptide segment around the modified site. The existed methods of PTM prediction often focus on the linear neighboring amino acid residues, which considered the piece-wise part of a small interval upstream or downstream of the modified site. However in the real case, the modified site often has not only the linear-contacted residue neighbors but also more neighboring amino acid residues which are linearly far away from the target site but actually contacted with it based on different spatial structures by various ways of peptide folding. There are plenty of studies attempting to solve the problem of predicting which amino acid residues in the structure are “in contact” with each other, which refers to the case that when the distance between their *β*-carbin (or *α*-carbin for the amino acid glycine) is smaller than 8 $\overset{{}^{\circ}}{A}$ Luttrell et al. (2019). In this paper, we considered the spatial residue–residue contact information into the structure of peptides and employed the spatial structure of residue–residue contact into the encoding of features. In this way, not only the linear segments around the modified site but also the spatial surrounding residues are considered for analysis of Kcr modification. This is the first time that spatial neighbored residues are considered into the encoding of features for PTM prediction, and we found that it can be an effective feature which could yield a promising good performance and has achieved performances as good as those generally used features.

## 2 Materials and Methods

### 2.1 Data Pre-processing

In this research, the steps proposed by Zhao, Y. et al. were followed to collect the experimentally verified Kcr sites Zhao et al. (2020). As the obtained data were of a fixed length, which are only a part of the whole peptide sequence and cannot provide enough information for predicting its spatial structure, BLAST was used for the alignment of a partial peptide onto the whole sequence from the existing database first; in this case, the corresponding whole peptide sequence was found out by selecting whose alignment result provided 100 identity values. The whole sequences were obtained and were used as input for the prediction of residue–residue contact. We have obtained 511 peptides (the whole sequence instead of partial segments) after the alignment; among these whole peptide sequences, there are totally 10,944 sites, consisting of 1,119 positive and 9,825 negative samples (they are slightly different in the sample numbers for datasets of different window size numbers; here are the sample numbers from the dataset which is of a window length of 10). The detailed datasets are provided in the supplementary materials. Among this dataset, 80 and 20% were randomly divided for the training and testing datasets, which gives 6,902 negative samples and 758 positive samples in the training dataset and 2,923 negative samples and 361 positive samples in the testing dataset. Since the dataset is very imbalanced with positive and negative samples of a ratio around 1:10, it would cause the performance of cross validation biased He and Garcia (2009). Because of this, the random under sampling method was employed onto the training step to make the positive and negative sites equal-sized in the training set, while the testing set keeps imbalanced for validation.

### 2.2 Overview of Our Prediction Model

There are two phases in this research. The first is residue–residue contact prediction. In this phase, a released tool named MapPred Wu et al. (2020) was employed for the prediction as it was of relatively higher performance in terms of both prediction accuracy and computation speed Wuyun et al. (2016). It also can be accessed via a web server, which is more user-friendly. The residue–residue contact prediction tool will provide a table containing six columns as a prediction result; the first and second columns are the indices of two amino acid residues in the whole peptide sequence, and the fifth column is the probability of which the two residues of the indices (in the first and second columns) are contacted. In this research, we chose 0.80 as a threshold value, which means that if the prediction probability value is higher than 0.8, we regarded these two residues as contacted, otherwise not. These selected contacted residues will be used in the later phase of feature representation.

**FIGURE 1:**
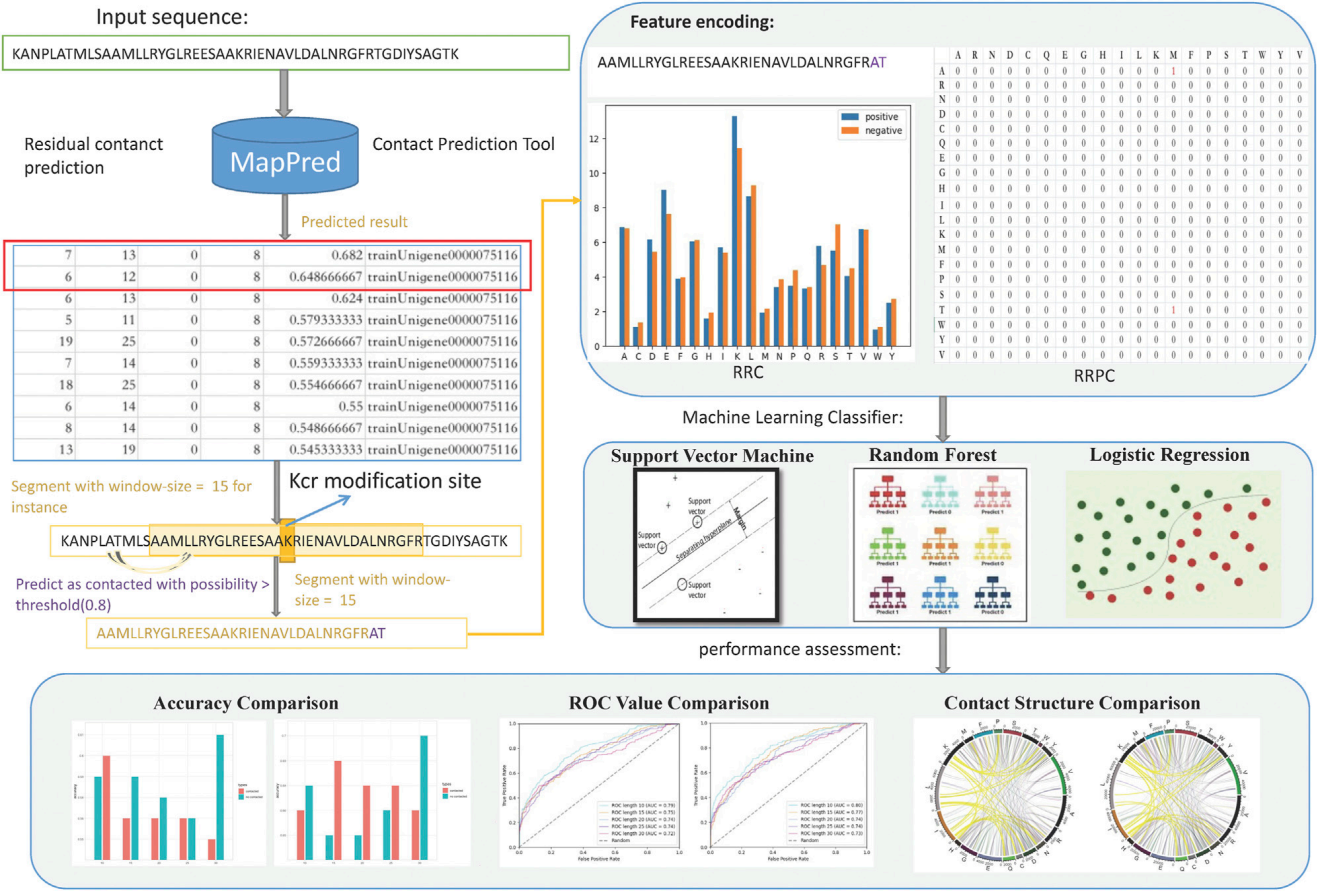
Flowchart of this paper; the main steps are data pre-processing, feature encoding, classification, and performance assessment. In data pre-processing, whole peptide sequences were used as inputs of a contact prediction tool, which gives a table-formed result, suggesting whether two residues (indices shown in column 1 and column 2) are contacted, and the possibility (in column five) that these two residues are contacted. Then, we append the contacted residues at the tail of the sequence segment and used the expanded segments for feature encoding, obtaining RRC and RRPC features. Then, machine learning methods were involved for Kcr prediction. In the performance assessment part, we compared the accuracy and AUC criterion between spatial and linear features; then, visualization of different window length segments was carried out.

The next phase is Kcr prediction. We applied the residue–residue contact information into the feature representation part; then, the procedure follows the general PTM prediction steps. We applied different machine learning classifiers including support vector machine (SVM), random forest (RF), and logistic regression (LR). Then, we carried out result assessment for each feature. For analyzing whether those spatially related features (RRC and RRPC) are efficient, we also applied feature selection based on the best-performed spatial feature together with other generally used features which are encoded based on linear segments such as binary encoding (BE), composition of k-spaced amino acid pairs (CKSAAP), enhanced amino acid composition (EAAC), enhanced group amino acid composition (EGAAC), and position-specific score matrix (PSSM). The flowchart of this paper is indicated in Figure 1.

### 2.3 Feature Representation

In this research, both linear features and new-raised spatial features were taken into consideration.

#### 2.3.1 Linear Feature

Linear features stand for the general-adopted features; these features are encoded with a piece of peptide of certain window size *n* upstream or downstream of the target amino acid site, which forms a segment of length 2∗*n* + 1. It contains only the neighbored amino acid composition and depends only on different window size numbers. In this paper, we have chosen a window size number equal to 10, 15, 20, 25, and 30 for linear feature encoding.

##### 2.3.1.1 AAC

AAC is the most basic and widely used feature in PTM prediction; it indicates the frequency that each type of amino acid occurs in a peptide. As there are 20 types of amino acids in a protein sequence, the dimension of an AAC feature is 20. For the sequence *x*, which is of fixed length *n* (n refers to the window size), the probability *P*_*x*_(*k*) of amino acid *k* is

$$P_{x}\left(k\right)=\frac{n_{x}\left(k\right)}{n}$$

where *n*_*x*_(*k*) refers to occurrence of amino acid *k*. In this paper, we encoded the AAC feature based on a window size of 10, 15, 20, 25, and 30 .

##### 2.3.1.2 AAPC

AAPC is the pair-wise feature of AAC as it indicates the frequency that each type of amino acid pair occurs in a peptide. There are totally 20 types of amino acids in protein; hence, 20∗20 types of amino acid pairs are available, so the dimension of the AAPC feature should be 400. The probability *P*_*x*_(*k*) of an amino acid pair in a sequence *x* is

$$P_{x}\left(k\right)=\frac{n_{x}\left(k\right)}{n*\left(n-1\right)}$$

where *n*_*x*_(*k*) is the occurrence of amino acid pair *k*. In this paper, we encoded the AAPC feature based on the same window size as the AAC feature, which is 10, 15, 20, 25, and 30 Wang et al. (2020).

#### 2.3.2 Spatial Feature

##### 2.3.2.1 RRC

**FIGURE 2:**
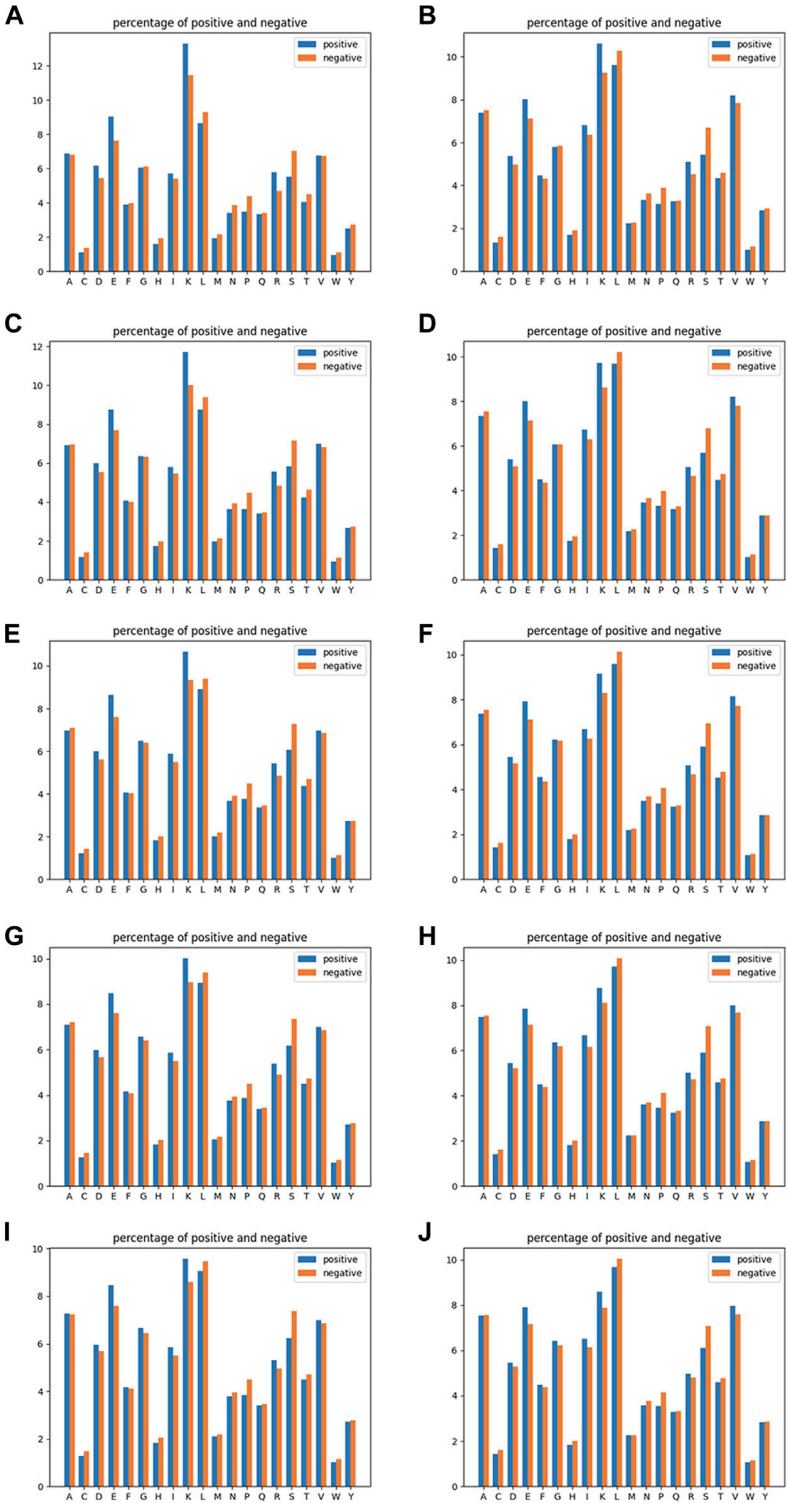
Amino acid composition analysis of linear feature AAC (the left-column figures) and spatial contacted feature RRC (the right-column figures). The five rows correspond to window sizes of 10, 15, 20, 25, and 30 from the top to the bottom. In detail, **(A)** AAC of a window size of 10, **(B)** RRC of a window size of 10, **(C)** AAC of a window size of 15, **(D)** RRC of a window size of 15, **(E)** AAC of a window size of 20, **(F)** RRC of a window size of 20, **(G)** AAC of a window size of 25, **(H)** RRC of a window size of 25, **(I)** AAC of a window size of 30, and **(J)** RRC of a window size of 30.

RRC is of a similar rule to AAC, but the residue–residue contact part has been taken into consideration. The residue–residue contact result obtained from residue–residue contact prediction was used here; if one of the contacted residue pair is within the range of *n* amino acids upstream and downstream of the target site, then we expand it to the range of the − *n* to + *n* segment (where 0 is the location of the target site), and then we used the same rule as AAC to compute the frequency of each amino acid. The dimension of RRC is the same as AAC, also 20. Similar to AAC, we chose n equal to 10, 15, 20, 25, and 30 for the residue–residue contact selection. The reason why we chose AAC as a basic rule for expanding a new spatial feature is that the dimension of the AAC feature is fixed as the total type of amino acid is of a fixed number, which means that the order we arrange these contacted residues would have an impact on neither the dimension nor the feature value in each dimension of feature RRC as we need not consider the order of residues but only their occurrence. The visualization of AAC and RRC is indicated in Figure 2.

##### 2.3.2.2 RRPC

**FIGURE 3:**
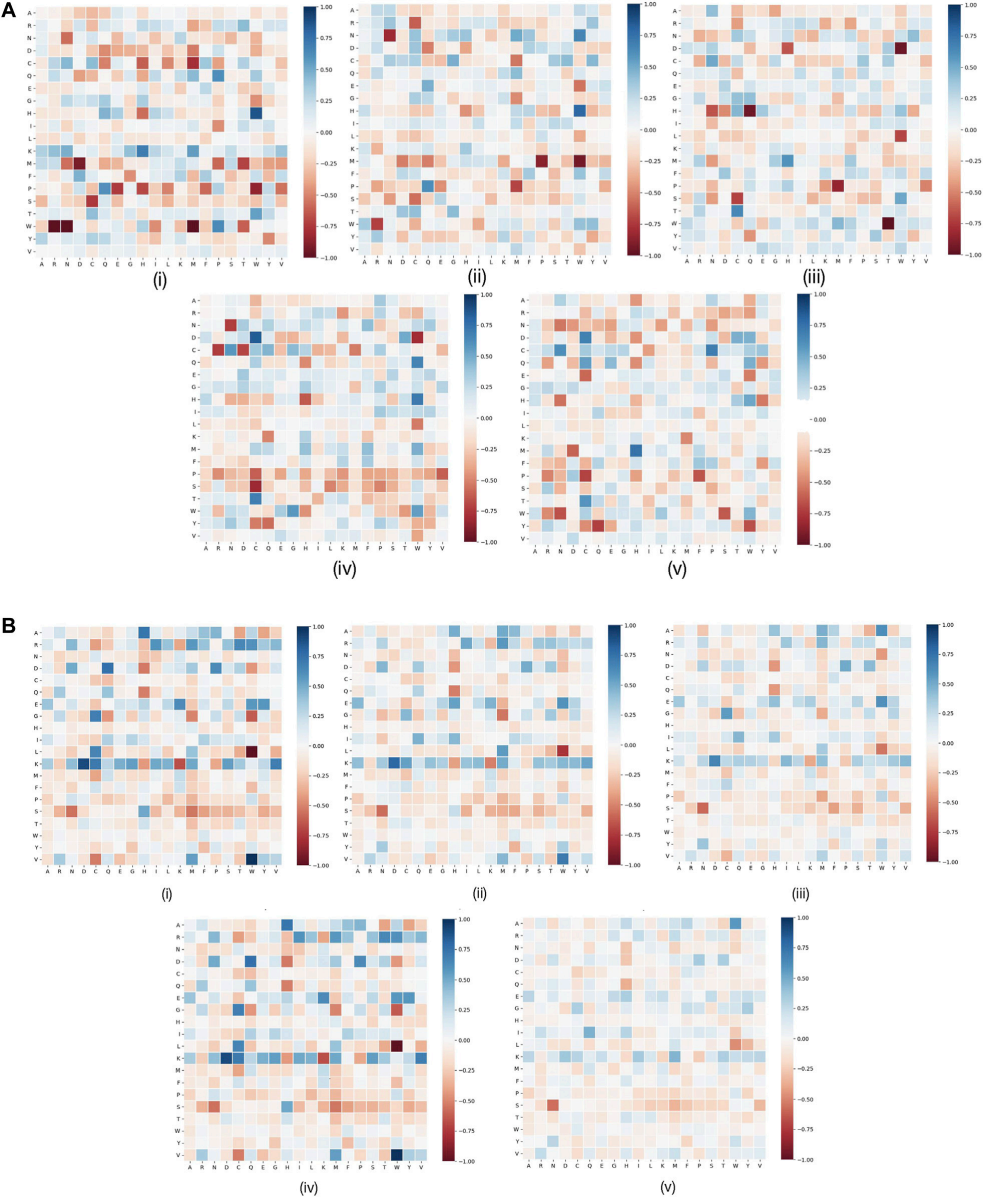
Visualization of features RRPC and AAPC. In detail, for Figure **(A)**, (i) RRPC of a window size of 10, (ii) RRPC of a window size of 15, (iii) RRPC of a window size of 20, (iv) RRPC of a window size of 25, and (v) RRPC of a window size of 30. For Figure **(B)**, (i) AAPC of a window size of 10, (ii) AAPC of a window size of 15, (iii) AAPC of a window size of 20, (iv) AAPC of a window size of 25, and (v) AAPC of a window size of 30.

RRPC is of a similar rule to the AAPC feature; in this feature, the residue–residue prediction result was directly used; that is, if the two residues are considered to be contacted, then we regarded it as one type of RRPC. The dimension is also 400 as there are overall 20∗20 = 400 types of possible combinations of residue–residue contact, which is of the same dimension number as AAPC. For the same reason that we encoded RRC based on AAC, the reason that we encoded RRPC based on AAPC is that the dimension of the AAPC feature is also fixed as the total type number of amino acid pairs is constant; in this case, we can make comparison between AAPC and RRPC as they are of a similar encoding method, and in this research, we assign residue–residue pairs based on the index order in ascending order. The visualization of RRPC is indicated in Figure 3.

### 2.4 Feature Selection

For the aim of improving prediction performance and removing redundant dimensions of the feature to speed up the prediction process, feature selection is a phase which is of paramount importance Guyon and Elisseeff (2003). In the feature selection procedure, each dimension of the feature vectors was ranked according to a certain criterion of “importance”; then, those of lower “importance” would be deleted, and then, the feature vector will be of a lower dimension but higher importance, which is more information-rich than the original encoding feature Tang et al. (2020). In this study, the data in the training sets were applied in the feature selection procedure and then follow the steps of validation and independent testing. The feature dimensions were ranked according to their merit information gain in IG, the value of the *χ* ^2^ statistic in the Chi-square method Chen et al. (2007).

#### 2.4.1 Chi-Square Method

In the ranking step of the Chi-square value method, the Chi-square value of each feature was calculated by

$$\chi^{2}=\overset{m}{\underset{i=1}{\sum }}\frac{{\left(n_{i}-np_{i}\right)}^{2}}{np_{i}}$$

where *n*_*i*_ is the number of instances, which will result in the outcome *x*_*i*_ Chen et al. (2007). A feature that gives a higher value of *χ* ^2^ receives a lower rank; then, according to the *χ* ^2^ value, the *p*-value for each dimension of the individual feature was obtained and the *p*-value greater than 0.05 was removed. This selection was taken on each feature for deleting those redundant dimensions.

### 2.4.2 Information Gain Method

For the information-gain method, it follows a similar procedure to the Chi-square method. It measures the entropy in descending order when a given feature is used to group values of another feature. The entropy of a feature *x* is defined as

$$H\left(x\right)=-\underset{i}{\sum }P\left(x_{i}\right)\log_{2}\left(P\left(x_{i}\right)\right)$$

where *x*_*i*_ is a set of values of *x* and *P*(*x*_*i*_) is the prior probability of *x*_*i*_. The conditional entropy of *x*, given another feature *Y*, is defined as

$$H\left(x|Y\right)=-\underset{j}{\sum }P\left(y_{j}\right)\underset{i}{\sum }P\left(x_{i}|Y_{j}\right)\log_{2}\left(P\left(x_{i}|Y_{j}\right)\right)$$

According to the entropy of *x* for a given *Y*, we ranked those features and deleted those redundant dimensions Chen et al. (2009).

### 2.5 Model Construction and Performance Evaluation

This study involves the machine learning method in the prediction of crotonylation. Support Vector Machine (SVM), Random Forest (RF), and Logistic Regression (LR) methods are adopted. We employed these three types of classifiers for Kcr prediction based on both linear features and spatial features and then compared the performance of the same type of classifier in the later part of performance analysis.

#### 2.5.1 Model Construction

##### 2.5.1.1 Support Vector Machine

As a classical machine learning method, SVM is the most-often-used method for classification problems which are of enough data but not as plenty as required for the deep learning method. It is a supervised learning method that was first proposed in 1963 by Vapnik, V. and Lerner, A.Y. in the field of pattern recognition Vapnik and Lerner (1963). After development in decades, it is still the top-used machine learning method in binary-class-division. SVM is based on associated learning algorithms using regression analysis to classify data Vapnik (1999); the main idea is to find out a boundary that can separate samples into different parts.

In this study, SVM with a radial basis function (RBF) kernel was adopted. Penalty parameter *C* was selected from set {2^0^, 2^1^, 2^2^, *…* , 2^10^}, and the kernel parameter *γ* was selected from set {2^–10^, 2^–9^, 2^–8^, *…* , 2^0^} by grid searching. The SVM classifier was developed by using the python module ‘sklearn’ Chen et al. (2020), Chen Z. et al. (2021).

##### 2.5.1.2 Random Forest

The RF method is another widely adopted method in the field of machine learning, which was first proposed in 2001 by Breiman, L Breiman (2001). It is a combination of tree predictors such that each tree depends on the values of a random vector sampled independently and with the same distribution for all trees in the forest. RF is more advanced than the traditional machine learning method as it can work efficiently in more complicated cases and gives out a more balanced result when imbalanced data have been provided. The training process of RF was by setting the tree number from set {1,400, 1,600, 1800, … , 2,400}, and it is also implemented based on the python module ‘sklearn’ Chen et al. (2020), Chen Z. et al. (2021).

##### 2.5.1.3 Logistic Regression

LR is a wildly adopted algorithm for binary classification, which consists of two main steps: training and regression. In the training phase, the weights and bias by using SGD and the cross-entropy loss function were employed, which makes an approximation of those samples, and then, in the regression phase, the approximated function was used for predicting whether those data are positive or negative. In this research, we set a cutoff value of 0.5. This classifier is also implemented based on the python module ‘sklearn’ Chen et al. (2020), Chen Z. et al. (2021).

##### 2.5.2 Performance Evaluation

In the phase of machine learning classification, k-fold cross-validation was employed to evaluate the predictive performances Kao et al. (2015). When implementing k-fold cross-validation, all the training data, including positive and negative sequences, were randomly clustered into k equal-sized subgroups. After that, k-1 of them shall be regarded as the training sample and the remaining one subgroup was considered as the validation sample. In a round of k-fold cross-validation, each of the k subgroups should be considered as the validation sample once in turn. In this study, k equal to 5 was chosen for the cross-validation Liu (2019).

Sensitivity (Sn), specificity (Sp), accuracy (Acc), Matthews correlation coefficient (MCC), Recall, Precision, and F1_Score have been used as the metrics to determine the performance of the generated models. The four metrics are defined in terms of TP, FN, TN, and FP, which denote the instances of true positive, false negative, true negative, and false positive, respectively, as Chen Z. et al. (2021), Liu et al. (2016)

$$\begin{array}{l} Sn=\frac{TP}{TP+FN},\\ Sp=\frac{TN}{TN+FP},\\ Acc=\frac{TP+TN}{TP+FP+TN+FN},\\ MCC=\frac{TP\times TN-FN\times FP}{\sqrt{\left(TP+FN\right)\times \left(TN+FP\right)\times \left(TP+FP\right)\times \left(TN+FN\right)}},\\ Recall=\frac{TP}{TP+FN}=Sn,\\ Precision=1-\frac{FP}{TP+FP},\\ F1\text{\_}\text{Score}=1-\frac{FP+FN}{2\times TP+FP+FN}. \end{array}$$

The ROC curve is also adopted as an evaluation criterion in this study as a more objective measurement than sensitivity and specificity. The area under curve (AUC) is an important criterion in performance evaluation for imbalanced cases Li et al. (2018). After evaluating the k-fold cross-validation, the classifier which achieved the best predictive performance was further evaluated by an independent testing dataset that was not included at all in the training samples.

Besides the performance evaluation of each single feature, the performance of the incorporated features which combined different features was also assessed by two feature selection (Chi-square and information-gain) methods.

## 3 Results and Discussion

**FIGURE 4:**
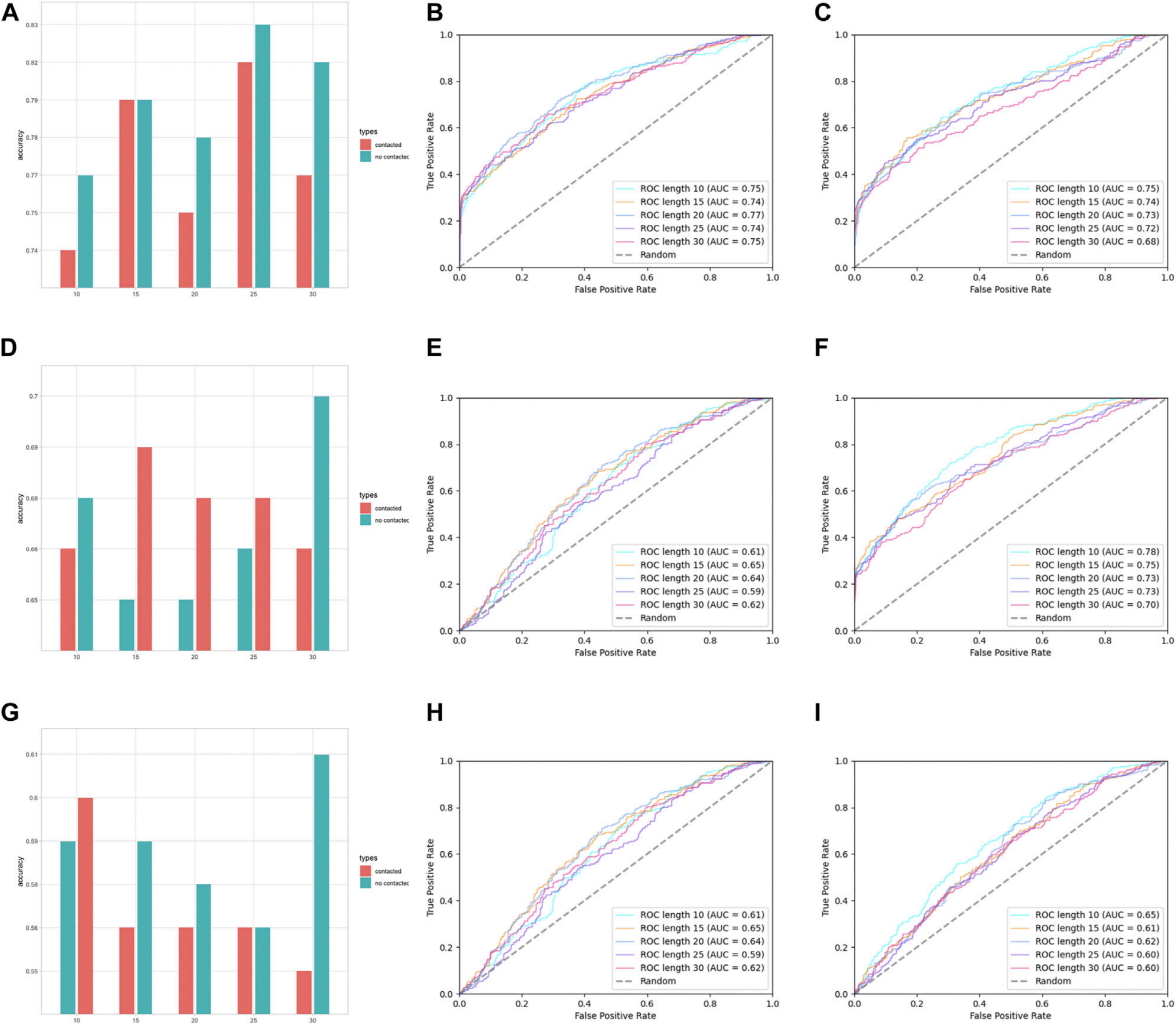
Performance assessment of features AAC and RRC. **(A)** Group barchart of classifier SVM of features AAC and RRC; **(B)** ROC of feature AAC with classifier SVM; **(C)** ROC of feature RRC with classifier SVM; **(D)** group barchart of classifier RF of features AAC and RRC; **(E)** ROC of feature AAC with classifier RF; **(F)** ROC of feature RRC with classifier RF; **(G)** group barchart of classifier LR of features AAC and RRC; **(H)** ROC of feature AAC with classifier LR; **(I)** ROC of feature RRC with classifier LR. In (a), (d), and (g), the red bars refer to contacted information, which corresponds to the RRC feature, while green bars refer to the case containing no contacted residues, which corresponds to AAC features.

In the assessment of different features, we have grouped AAC and RRC into one group, labeled as the residue composition feature; AAPC and RRPC were grouped into the other group for comparison of performances, and we labeled these two features as residue–residue pair-wise features. The performances of group residue composition features are listed in Suppplementary Table S1 in Supplementary Materials, and the visualization of the performance is attached in Figure 4.

In Figure 4A–C, it can be seen that the performances of features AAC and RRC are competitively good with classifier SVM (the performance of AAC is slightly higher); similarly in Figure 4G–I for the case of LR classifier, feature AAC achieved a slightly higher accuracy than feature RRC expect the case when the window size is 10. Surprisingly, in the case of classifier RF (Figure 4D–F) with different window-size numbers, feature RRC yielded a higher accuracy and a better overall AUC value, which means that in the classifier RF, the feature RRC could achieved a performance with not only better accuracy but also a higher AUC than the feature AAC, which means that in the residue composition feature, the spatial feature could yield a competitively good or even better performance than the traditional linear feature. However, overall, the performance of RRC is not obviously higher than that of AAC, which is reasonable as in Figure 2, it shows that for RRC, it seems that less difference appears between positive and negative, which is reasonable as for RRC, we considered more residues into one segment, which would give higher occurrence of each type of amino acid when the window size becomes larger. This makes it explainable that the difference between positive and negative samples becomes smaller when the window size numbers go larger as when a larger window size is given, the effect those contacted residues have on the whole segment for feature encoding would be less.

**FIGURE 5:**
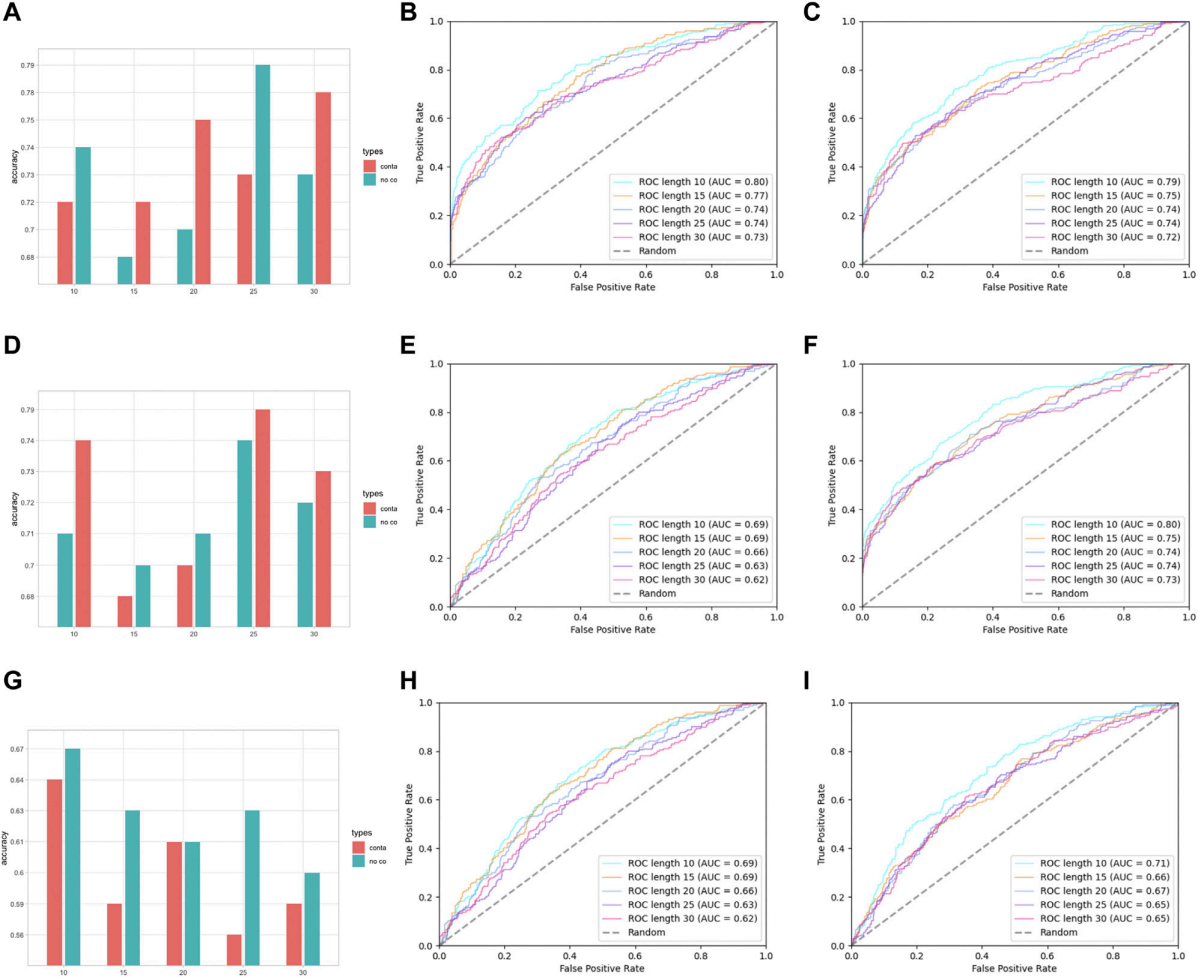
Performance assessment of features AAPC and RRPC. **(A)** Group barchart of classifier SVM of features AAPC and RRPC; **(B)** ROC of feature AAPC with classifier SVM; **(C)** ROC of feature RRPC with classifier SVM; **(D)** group barchart of classifier RF of features AAPC and RRPC; **(E)** ROC of feature AAPC with classifier RF; **(F)** ROC of feature RRPC with classifier RF; **(G)** group barchart of classifier LR of features AAPC and RRPC; **(H)** ROC of feature AAPC with classifier LR; **(I)** ROC of feature RRPC with classifier LR. In **(A)**, **(D)**, and **(G)**, the red bars refer to contacted information, which corresponds to the RRC feature, while green bars refer to the case containing no contacted residues, which corresponds to AAC features.

Except for residue composition features, residue–residue pair-wise features were also considered; here, the performances of pair-wise features AAPC and RRPC are attached in Suppplementary Table S2 in Supplementary Materials and Figure 5.

In Figure 5, it shows that for pairwise features, higher improvement of performances in feature RRPC appeared, with the case of classifier SVM and RF, and in most of the cases with different window size numbers, feature RRPC yielded higher accuracy and AUC values. In the SVM classifier (Figure 5A–C), the accuracy of feature RRPC (in Figure 5A) is obviously higher than of feature AAPC, while the AUC values(Figure 5B, C for AAPC and for RRPC) are competitively high. Meanwhile, in the classifier of RF (Figure 5D–F), the RRPC feature gives not only a better accuracy but also an obvious overall AUC value when the window size changes. In the LR classifier (Figure 5G–I), the performances of various window size numbers are very close in features AAPC and RRPC, with similarly good accuracy and AUC. Comparing with the single-amino-acid-related feature, the performances in the pair-wise feature are more outstanding and robust, which may be owned by the reason that the differences between positive and negative samples in the pair-wise feature are more obvious than the single-amino-acid-related feature. As indicated in Figure 2, the differences between percentages of positive and negative are not quite various when the window size changes, and the value differences between positive and negative samples are not large in a fixed window size. However, in Figure 3, it shows great differences among positive and negative samples, and larger differences appeared in a smaller number of window size as it shows more plain color in the feature AAPC. This gives the evidence that spatial-factor-related feature RRPC could be a more reliable feature for Kcr prediction as larger differences indicated on it than feature AAPC.

Besides, we have combined the features RRC and RRPC into an incorporated feature and compared the performance with incorporated AAC and AAPC. The detailed performance has been indicated in Suppplementary Table S3 in Supplementary Materials. In this case, the performance of incorporated RRC and RRPC is also similarly high as incorporated AAC and AAPC, which shows that the spatial features RRC and RRPC are both efficient in the classification of Kcr sites in both single features and incorporated features. Among these two, RRPC tends to be more efficient in the smaller-window-size case as it shows relatively higher performance than RRC or AAPC.

**FIGURE 6:**
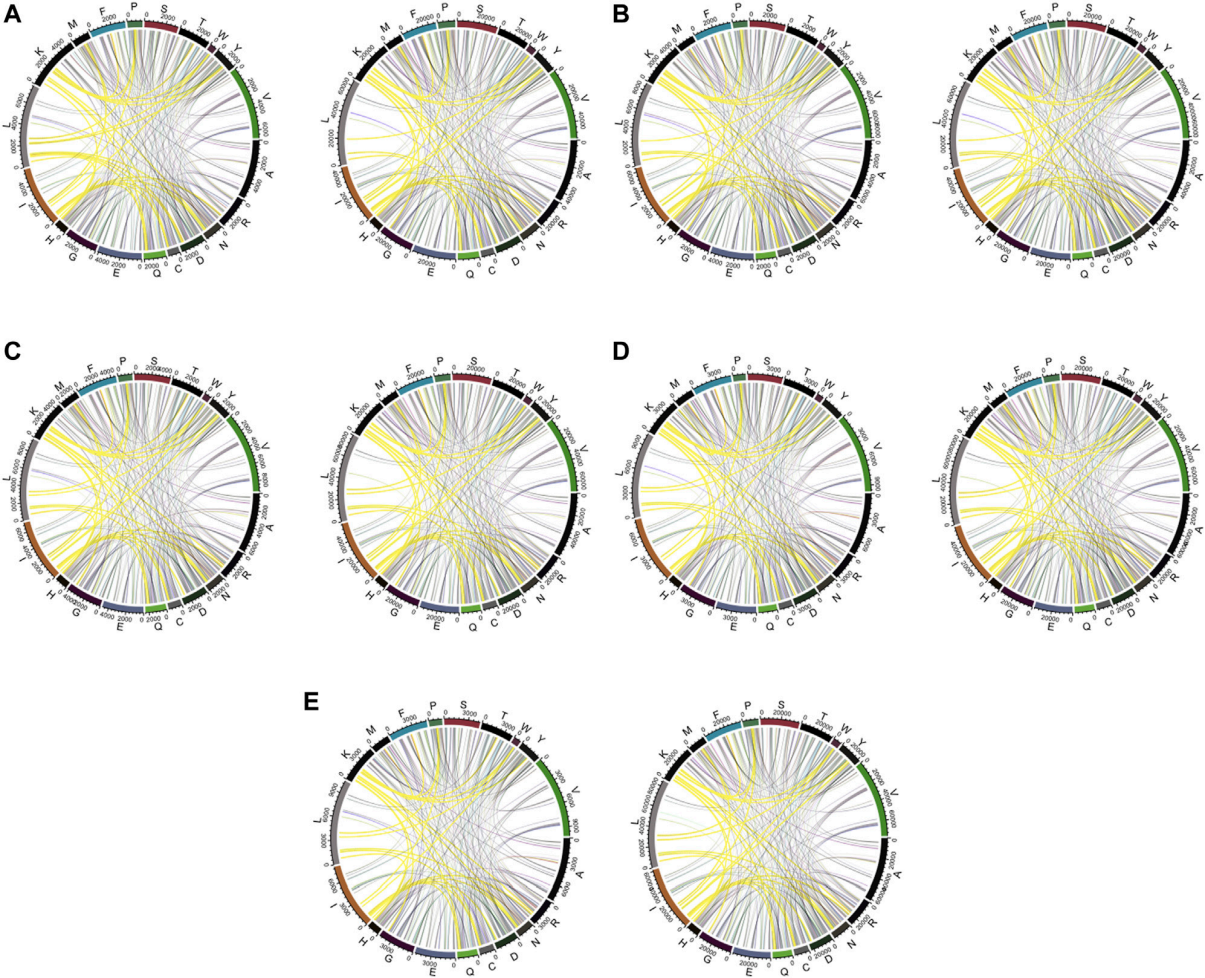
Chord diagram of a window size of **(A)** 10, **(B)** 15, **(C)** 20, **(D)** 25, and **(E)** 30. The linked lines show the contact between residues. Highlighted contacts are compared among various window sizes. For each sub-graph, the left one is from positive samples and the right one from negative samples.

For further discussion of residue–residue composition contact within each type of amino acid, the visualization of amino acid contact is generated in Figure 6. We used links to indicate the contact between residue and residue. As shown in Figure 6, it tends to be more pairs of Lx (residue L and other amino acids) in positive data when the window size is relatively small, typically in Figure 6A. When the window size becomes larger, the pair number of Lx for positive and negative sets tends to be more close to each. This is the reason why when we visualize the RRPC pair, in the case of a window size equal to 10, we obtained the result with the largest color range, and when the window size increases, the color range becomes more and more centralized to zero level and gives more pale color in the figure. It also suggests that when a smaller window size is considered, the effects of spatial residue contact are higher, which may explain why the performance of a smaller window size tends to be better than the larger-window-size case.

Feature selection was carried out for finding the ‘importance’ of each feature as well. We have selected features of cases with and without RRPC involved, and the selection result is attached in Supplementary Figures S4–7 in Supplementary Materials and the result of incorporated some sequence-based features with or without RRPC is attached in Table.1.

**TABLE 1:** Performance of incorporated features in the cases with or without RRPC incorporated. Here, the sequence-based features used for incorporation contain Binary, CKSAAP (k = 1, 2, 3), EAAC, EGAAC, and PSSM.

Feature	Classifier	Sensitivity	Specificity	Accuracy	MCC	Recall	Precision	F1-score
incorporated_without_RRPC	SVM	0.70	0.69	0.69	0.30	0.70	0.31	0.43
	RF	0.72	0.80	0.79	0.43	0.72	0.42	0.53
	LR	0.66	0.66	0.66	0.24	0.66	0.28	0.39
incorporated_with_RRPC	SVM	0.67	0.70	0.70	0.29	0.67	0.31	0.43
	RF	0.75	0.80	0.79	0.46	0.75	0.43	0.55
	LR	0.68	0.66	0.67	0.26	0.68	0.29	0.40

**FIGURE 7:**
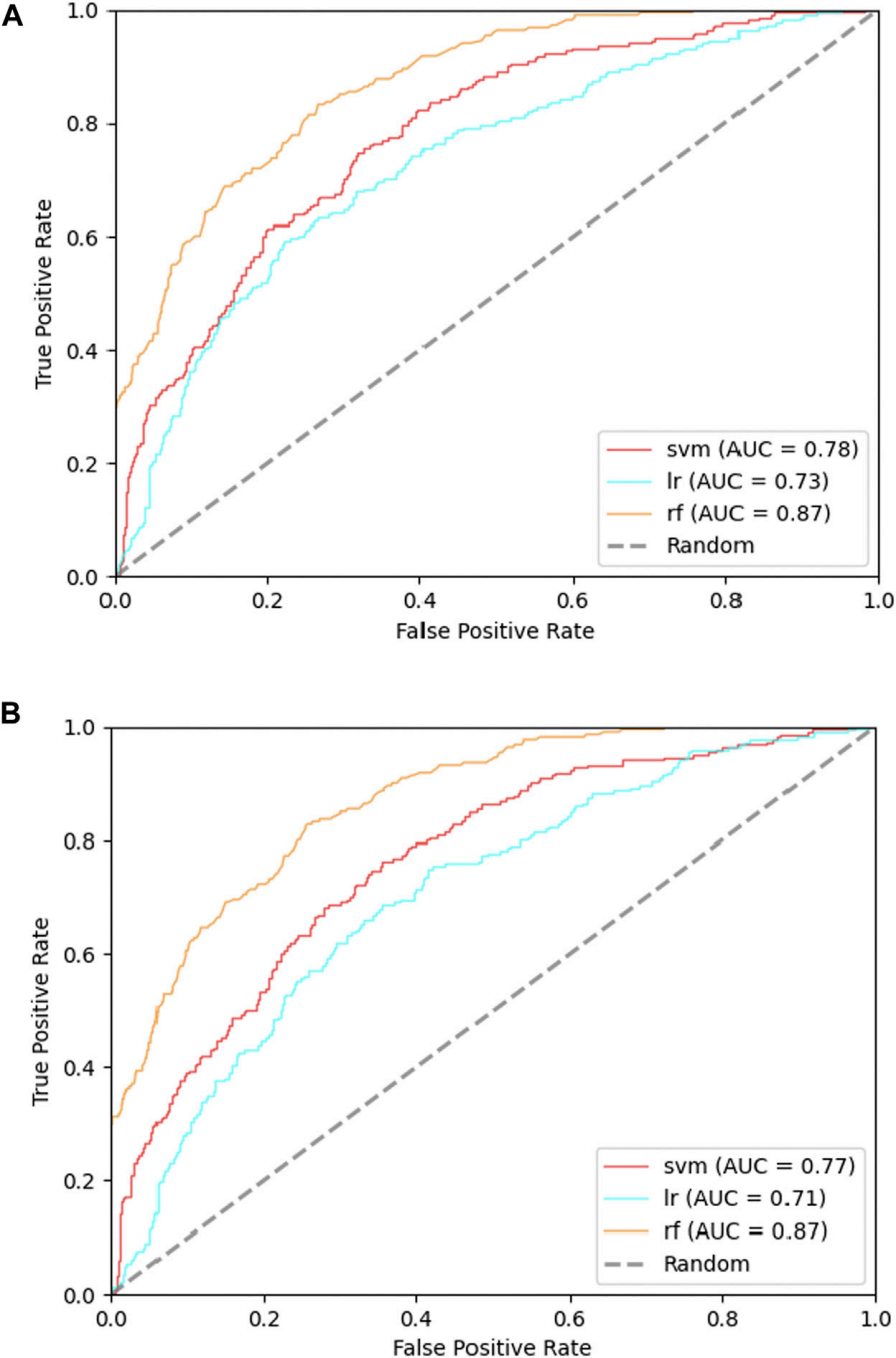
ROC curves of incorporated features in the cases with or without RRPC incorporated: **(A)** incorporated with RRPC and **(B)** incorporated without RRPC.

In order to find whether the feature RRPC is of enough importance for Kcr prediction, we separated the feature selection part into two cases: one is selection with RRPC involved, and the other one is without RRPC involved. Each of these two cases has been applied Chi-square selection and information-gain selection. The result is shown in Figure 7. Compared with Figure 7B, which shows the result of the Chi-square method selection without RRPC contains, the performance of classifier SVM has increased 0.2, while the other two are with 0.1 lower. However, for the information-gain selection method, the performance based on an incorporated feature without RRPC when SVM employed is 0.3 higher than the case incorporated with RRPC, while other two are equivalently good (for the RF classifier) and better if incorporated with RRPC (LR classifier). Overall, the features incorporated with RRPC can yield a competitively good or even higher performance compared to the features without RRPC combined, and as shown in the selection result, we can see that the RRPC feature has taken a large percentage when selecting, which proves that RRPC is an efficient feature which is of high importance and good performance as well. The table-formed result assessments have been attached in Suppplementary Tables S4, 5 in Supplementary Materials.

## 4 Conclusion

In this research, we applied information of spatial residue–residue contact into encoding of features, represented as RRC and RRPC, for classification of modified Kcr sites and compared with other sequence-based features on the aspects of accuracy, AUC values, and other criteria for performance assessment. It shows that RRC and RRPC can be effective in classifying Kcr sites as the performance can be as competitively good as other well-known features such as AAC, AAPC, CKSAAP, etc. We also employed feature selection together with the RRPC feature as it yielded a better performance than RRC and shows that RRPC has taken a large weight of selected features, which shows that RRPC is of high information-rich properties and efficient enough for the Kcr prediction. However, there are some more details that require further research: the first one is that the prediction step of residue–residue contact is very time-consuming; in Suppplementary Tables S6, 7 of Supplementary Materials, a summary of some recent-released tools for residue–residue contact prediction and the comparison of our adopted tool in this research and other tools are attached, it can be noticed that even the most speedy tool requires plenty of time for computation and not a few of them gave the final prediction result within 24 h, which is very time-consuming and makes it challenging to broaden the application of residue–residue contact into the encoding scheme. Another problem is that we can see that for Kcr modification, the best performances of spatial features are of a similar level or slightly higher compared with traditional linear features, but for different types of PTM, the surrounding residues of modified sites might be different as each type of PTM requires a certain environment for modification; also, for different PTM, it suggests different types of modifications, which suggests that different effects may be of different PTM when residue–residue contact is considered to be a feature, which is another approach that requires discussion.

### 4.1 Permission to Reuse and Copyright

Figures, tables, and images will be published under a Creative Commons CC-BY licence, and permission must be obtained for use of copyrighted material from other sources (including re-published/adapted/modified/partial figures and images from the internet). It is the responsibility of the authors to acquire the licenses, to follow any citation instructions requested by third-party right holders, and to cover any supplementary charges.

## Data Availability

The original contributions presented in the study are included in the article/Supplementary Material; further inquiries can be directed to the corresponding author.
