# Supplementary material for: Residue–Residue Contact Can Be a Potential Feature for the Prediction of Lysine Crotonylation Sites
Source: Front Genet. 2022 Jan 4;12:788467. doi: 10.3389/fgene.2021.788467 (PMC8764140; doi:10.3389/fgene.2021.788467)
Supplement: Supplementary file 1 [file DataSheet1.PDF]

Classifier	Window size	Feature name	Sn	Sp	Acc	MCC	Recall	Precision	F1-score
SVM	10	RRC	0.57	0.78	0.74	0.29	0.57	0.34	0.43
		AAC	0.51	0.83	0.77	0.3	0.51	0.37	0.43
	15	RRC	0.54	0.84	0.79	0.34	0.54	0.4	0.46
		AAC	0.44	0.86	0.79	0.3	0.44	0.4	0.42
	20	RRC	0.55	0.79	0.75	0.29	0.55	0.35	0.43
		AAC	0.56	0.83	0.78	0.34	0.56	0.39	0.46
	25	RRC	0.45	0.89	0.82	0.35	0.45	0.46	0.45
		AAC	0.42	0.91	0.83	0.36	0.42	0.49	0.46
	30	RRC	0.45	0.84	0.77	0.26	0.45	0.35	0.4
		AAC	0.46	0.89	0.82	0.35	0.46	0.45	0.46
RF	10	RRC	0.62	0.67	0.66	0.22	0.62	0.27	0.38
		AAC	0.7	0.67	0.68	0.29	0.7	0.3	0.42
	15	RRC	0.61	0.7	0.69	0.24	0.61	0.29	0.39
		AAC	0.68	0.65	0.65	0.25	0.68	0.28	0.4
	20	RRC	0.66	0.68	0.68	0.26	0.66	0.29	0.4
		AAC	0.73	0.64	0.65	0.28	0.73	0.29	0.41
	25	RRC	0.61	0.7	0.68	0.24	0.61	0.29	0.39
		AAC	0.65	0.66	0.66	0.23	0.65	0.27	0.39
	30	RRC	0.62	0.67	0.66	0.22	0.62	0.27	0.38
		AAC	0.64	0.71	0.7	0.27	0.64	0.31	0.41
LR	10	RRC	0.62	0.6	0.6	0.16	0.62	0.23	0.34
		AAC	0.55	0.6	0.59	0.12	0.55	0.22	0.31
	15	RRC	0.58	0.56	0.56	0.11	0.58	0.21	0.31
		AAC	0.65	0.58	0.59	0.17	0.65	0.24	0.35
	20	RRC	0.62	0.54	0.56	0.12	0.62	0.21	0.32
		AAC	0.68	0.56	0.58	0.18	0.68	0.24	0.35
	25	RRC	0.56	0.55	0.56	0.09	0.56	0.2	0.3
		AAC	0.57	0.56	0.56	0.1	0.57	0.21	0.3
	30	RRC	0.61	0.54	0.55	0.11	0.61	0.21	0.31
		AAC	0.55	0.62	0.61	0.13	0.55	0.23	0.32

Table s1: Performance of RRC and AAC for fixed window size on different classifier. The result suggests that in classifier SVM, both RRC and AAC are of biased result with relatively low Sn but high Sp caused by the imbalancement of dataset. In RF, the result is balance and RRC gives better results overall(i.e., when window size equals to 15, 20 and 25). For LR classifier, both of these two features give a relatively lower performance than the other two, and AAC achieved a slightly better result than RRC in this case.

Classifier	Window size	Feature name	Sn	Sp	Acc	MCC	Recall	Precision	F1-score
SVM	10	RRPC	0.72	0.72	0.72	0.34	0.72	0.34	0.46
		AAPC	0.67	0.75	0.74	0.33	0.67	0.35	0.46
	15	RRPC	0.58	0.74	0.72	0.26	0.58	0.31	0.41
		AAPC	0.69	0.68	0.68	0.28	0.69	0.30	0.42
	20	RRPC	0.57	0.78	0.75	0.29	0.57	0.34	0.43
		AAPC	0.62	0.71	0.70	0.26	0.62	0.30	0.41
	25	RRPC	0.59	0.75	0.73	0.28	0.59	0.32	0.42
		AAPC	0.50	0.84	0.79	0.32	0.50	0.39	0.44
	30	RRPC	0.50	0.84	0.78	0.31	0.50	0.38	0.44
		AAPC	0.50	0.84	0.79	0.32	0.50	0.39	0.44
RF	10	RRPC	0.71	0.71	0.71	0.33	0.71	0.33	0.45
		AAPC	0.68	0.71	0.71	0.31	0.68	0.32	0.44
	15	RRPC	0.64	0.71	0.70	0.28	0.64	0.31	0.42
		AAPC	0.61	0.72	0.70	0.26	0.61	0.30	0.41
	20	RRPC	0.65	0.72	0.71	0.29	0.65	0.31	0.42
		AAPC	0.65	0.72	0.71	0.29	0.65	0.31	0.42
	25	RRPC	0.59	0.76	0.73	0.28	0.59	0.33	0.42
		AAPC	0.56	0.77	0.74	0.28	0.56	0.33	0.42
	30	RRPC	0.60	0.73	0.71	0.27	0.60	0.31	0.41
		AAPC	0.62	0.74	0.72	0.28	0.62	0.32	0.42
LR	10	RRPC	0.66	0.64	0.64	0.22	0.66	0.27	0.38
		AAPC	0.58	0.68	0.67	0.20	0.58	0.27	0.37
	15	RRPC	0.60	0.59	0.59	0.14	0.60	0.22	0.33
		AAPC	0.66	0.63	0.63	0.22	0.66	0.26	0.37
	20	RRPC	0.62	0.61	0.61	0.17	0.62	0.24	0.35
		AAPC	0.61	0.62	0.61	0.17	0.61	0.24	0.35
	25	RRPC	0.56	0.55	0.56	0.09	0.56	0.20	0.30
		AAPC	0.51	0.66	0.63	0.13	0.51	0.23	0.32
	30	RRPC	0.65	0.58	0.59	0.17	0.65	0.24	0.35
		AAPC	0.59	0.60	0.60	0.14	0.59	0.23	0.33

Table s2: Performance of RRPC and AAPC for fixed window size on different classifier. As the result indicates, most of the case with various classifiers, RRPC gives a better result than AAPC(i.e., window size equals 15, 20, and 30 in SVM, window size equals 10, 20 and 30 in RF) in classifier SVM and RF. In LR, these two features give very close performances.

Classifier	window size	Feature name	Sn	Sp	Acc	MCC	Recall	Precision	F1-score
SVM	10	spatial	0.64	0.78	0.75	0.34	0.64	0.36	0.46
		linear	0.72	0.72	0.72	0.34	0.72	0.34	0.46
	15	spatial	0.58	0.74	0.72	0.26	0.58	0.31	0.41
		linear	0.66	0.73	0.71	0.30	0.66	0.32	0.43
	20	spatial	0.57	0.78	0.75	0.29	0.57	0.34	0.43
		linear	0.58	0.79	0.75	0.31	0.58	0.35	0.44
	25	spatial	0.59	0.75	0.73	0.28	0.59	0.32	0.42
		linear	0.53	0.80	0.75	0.28	0.53	0.34	0.42
	30	spatial	0.50	0.84	0.78	0.31	0.50	0.38	0.43
		linear	0.50	0.86	0.80	0.34	0.50	0.42	0.45
RF	10	spatial	0.50	0.84	0.78	0.31	0.50	0.38	0.43
		linear	0.74	0.72	0.73	0.36	0.74	0.35	0.48
	15	spatial	0.65	0.72	0.71	0.29	0.65	0.32	0.43
		linear	0.63	0.71	0.70	0.27	0.63	0.30	0.41
	20	spatial	0.64	0.71	0.70	0.28	0.64	0.31	0.42
		linear	0.67	0.72	0.71	0.31	0.67	0.33	0.44
	25	spatial	0.59	0.75	0.73	0.28	0.59	0.33	0.42
		linear	0.57	0.79	0.75	0.30	0.57	0.35	0.43
	30	spatial	0.61	0.73	0.71	0.27	0.61	0.31	0.41
		linear	0.57	0.79	0.75	0.30	0.57	0.35	0.43
LR	10	spatial	0.67	0.64	0.64	0.23	0.67	0.27	0.38
		linear	0.58	0.66	0.65	0.19	0.58	0.26	0.36
	15	spatial	0.60	0.59	0.59	0.14	0.60	0.23	0.33
		linear	0.66	0.58	0.60	0.18	0.66	0.24	0.35
	20	spatial	0.62	0.61	0.61	0.17	0.62	0.24	0.34
		linear	0.63	0.60	0.60	0.17	0.63	0.24	0.35
	25	spatial	0.61	0.62	0.62	0.18	0.61	0.24	0.35
		linear	0.55	0.60	0.60	0.12	0.55	0.22	0.31
	30	spatial	0.65	0.58	0.59	0.17	0.65	0.24	0.35
		linear	0.61	0.57	0.57	0.13	0.61	0.22	0.32

Table s3: Performance of incorporated features. Spatial refers to the incorporation of two spatial-based features, which means RRC combined with RRPC, while linear refers to the incorporation of the sequence-based features, which means AAC combined with AAPC. As the result indicates, most of the case with various classifiers, RRPC gives a better result than AAPC(i.e., window size equals 15, 20, and 30 in SVM, window size equals 10, 20 and 30 in RF) in classifier SVM and RF. In LR, these two features give very close performances.

feature	classifier	Sensitivity	Specificity	Accuracy	MCC	Recall	Precision	F1-score
AAC	SVM	0.51	0.83	0.77	0.3	0.51	0.37	0.43
AAPC		0.67	0.75	0.74	0.33	0.67	0.35	0.46
BINARY		0.62	0.80	0.77	0.36	0.62	0.39	0.48
CKSAAP		0.63	0.72	0.71	0.28	0.63	0.31	0.42
EAAC		0.74	0.77	0.77	0.41	0.74	0.40	0.52
EGAAC		0.75	0.78	0.77	0.42	0.75	0.40	0.52
PSSM		0.47	0.86	0.80	0.32	0.47	0.42	0.44
RRPC		0.72	0.72	0.72	0.34	0.72	0.34	0.46
RRC		0.57	0.78	0.74	0.29	0.57	0.34	0.43
AAC	RF	0.7	0.67	0.68	0.29	0.7	0.3	0.42
AAPC		0.68	0.71	0.71	0.31	0.68	0.32	0.44
BINARY		0.69	0.73	0.72	0.33	0.69	0.34	0.45
CKSAAP		0.69	0.70	0.70	0.30	0.69	0.31	0.43
EAAC		0.73	0.77	0.76	0.40	0.73	0.39	0.51
EGAAC		0.76	0.79	0.78	0.44	0.76	0.41	0.53
PSSM		0.69	0.75	0.74	0.36	0.70	0.36	0.47
RRPC		0.71	0.71	0.71	0.33	0.71	0.33	0.45
RRC		0.62	0.67	0.66	0.22	0.62	0.27	0.38
AAC	LR	0.62	0.6	0.6	0.16	0.62	0.23	0.34
AAPC		0.58	0.68	0.67	0.20	0.58	0.27	0.37
BINARY		0.67	0.65	0.65	0.24	0.67	0.27	0.39
CKSAAP		0.56	0.68	0.66	0.19	0.56	0.26	0.36
EAAC		0.69	0.67	0.67	0.28	0.69	0.30	0.41
EGAAC		0.63	0.67	0.67	0.23	0.63	0.28	0.39
PSSM		0.67	0.64	0.64	0.23	0.67	0.27	0.39
RRPC		0.66	0.64	0.64	0.22	0.66	0.27	0.38
RRC		0.62	0.6	0.6	0.16	0.62	0.23	0.34

Table s4: Performance of general-adopted features and residue-residue contact based features on different classifiers. In this table window size was chosen to be 10 for feature encoding.

Method	Classifier	Feature	Sensitivity	Specificity	Accuracy	MCC	Recall	Precision	F1-score
CHI2	SVM	without_RRPC	0.68	0.69	0.69	0.29	0.68	0.31	0.42
		with_RRPC	0.76	0.73	0.74	0.39	0.76	0.36	0.49
	RF	without_RRPC	0.76	0.77	0.77	0.42	0.76	0.39	0.5
		with_RRPC	0.78	0.78	0.78	0.45	0.78	0.42	0.54
	LR	without_RRPC	0.62	0.68	0.67	0.23	0.62	0.28	0.38
		with_RRPC	0.68	0.69	0.69	0.3	0.69	0.31	0.43
IG	SVM	without_RRPC	0.71	0.7	0.7	0.32	0.71	0.32	0.45
		with_RRPC	0.65	0.69	0.68	0.26	0.65	0.29	0.4
	RF	without_RRPC	0.76	0.77	0.77	0.43	0.76	0.4	0.53
		with_RRPC	0.78	0.77	0.77	0.43	0.78	0.4	0.53
	LR	without_RRPC	0.67	0.7	0.68	0.28	0.67	0.3	0.42
		with_RRPC	0.67	0.69	0.69	0.28	0.67	0.3	0.42

Table s5: Performance of incorporated features in the cases of with or without RRPC incorporated.

Method	Class	Proposed year	Journal	Tool link	Availability	Type	Exceution time
SVMSEQ	1	2008	Bioinformatics	https://zhanglab.ccmb.med.umich.edu/SVMSEQ/	yes	webserver	more than 24 hours
NNcon	1	2009	Nucleic Acids Research	http://sysbio.rnet.missouri.edu/multicom_toolbox/tools.html#license	yes	toolbox	-
SVMcon	1	2007	Bioinformatics	http://sysbio.rnet.missouri.edu/multicom_toolbox/tools.html#license	yes	toolbox	-
PSpro (or PSpro.beta)	1	2012	BMC Bioinformatics	http://sysbio.rnet.missouri.edu/multicom_toolbox/tools.html#license	yes	toolbox	-
BETAcon	1	2005	Bioinformatics	http://sysbio.rnet.missouri.edu/multicom_toolbox/tools.html#license	yes	toolbox	-
bbcontacts	1	2015	Bioinformatics	http://github.com/soedinglab/bbcontacts	yes	toolbox	-
DNcon	1	2012	Bioinformatics	http://sysbio.rnet.missouri.edu/multicom_toolbox/tools.html#license	yes	toolbox	-
PSICOV	2	2012	Bioinformatics	http://bioinf.cs.ucl.ac.uk/downloads/PSICOV	yes	toolbox	-
CCMpred	2	2014	Bioinformatics	https://github.com/soedinglab/ccmpred	yes	toolbox	-
FreeContact	2	2014	BMC Bioinformatics	http://rostlab.org/owiki/index.php/FreeContact	yes	toolbox	-
GREMLIN	2	2013	Proc Natl Acad Sci USA	http://gremlin.bakerlab.org/index.php	yes	toolbox	-
plmDCA	2	2013	Phys Rev E Stat Nonlin Soft Matter Phys	http://plmdca.csc.kth.se/	yes	toolbox	-
MetaPSICOV	3	2015	Bioinformatics	http://bioinf.cs.ucl.ac.uk/psipred/	yes	webserver	2 hours and 19 minutes
PconsC2	3	2014	PLoS Comput Biol	http://pconsc3.bioinfo.se/	yes	webserver	more than 24 hours

Table s6: Comparison of some published residue-residue contact tools. In the column "Class",different numbers refer to different computation methods(i.e.: 1: machine learning based method, 2: direct-coupling-based methods and 3: direct-coupling-based methods, which means machine learning method incorporated with direct-coupling-based method). All the information above is from *Wuyun, Qiqige, et al. "A large-scale comparative assessment of methods for residue–residue contact prediction." Briefings in bioinformatics 19.2 (2018): 219-230.* Among these methods, we chose those with webservers and used a sequences of length more than 400 for computation speed test.

Method	SPOT-228	CAMEO-41	CASP12-38
R2C	37.66	NA	NA
DeepConPreda	38.9	NA	NA
CCMpred	46.9	45.85	38.22
NeBcon	48.88	NA	NA
MetaPSICOV	50.07	NA	NA
DNCON2	61.26	NA	NA
DeepCov	69.04	67.21	63.54
MapPred	78.94	77.06	**77.05**
RaptorX-Contact	78.3	**81.57**	75.8
SPOT-Contact	**81.66**	79.65	68.55

Table s7: Comparison between the precisions (%) of MapPred and other methods. This table is from *Wu, Qi, et al. "Protein contact prediction using metagenome sequence data and residual neural networks." Bioinformatics 36.1 (2020): 41-48.* The highest values are highlighted in bold. In this table it shows that MapPred is of higher performance than MetaPSICOV, can also competitively speedy from our other test as MapPred also gives the prediction result within 2 hours.

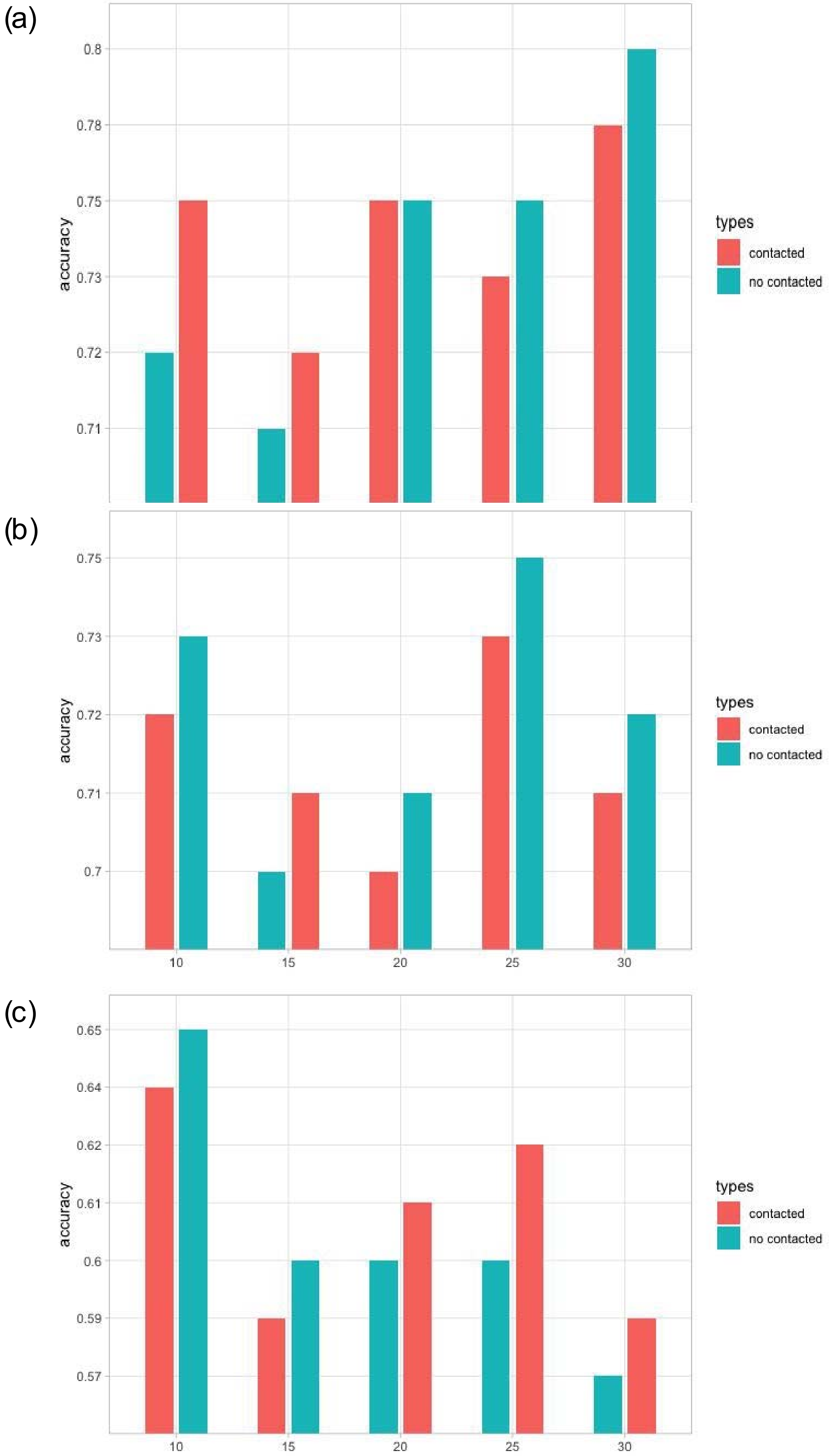

Figure s1: Grouped barcharts of incorporated features, in legend, types “contacted” means incorporation of RRC and RRPC, “no contacted” means incorporation of AAC and AAPC. (a). in SVM classifier, (b). RF classifier, (c) LR classifier.

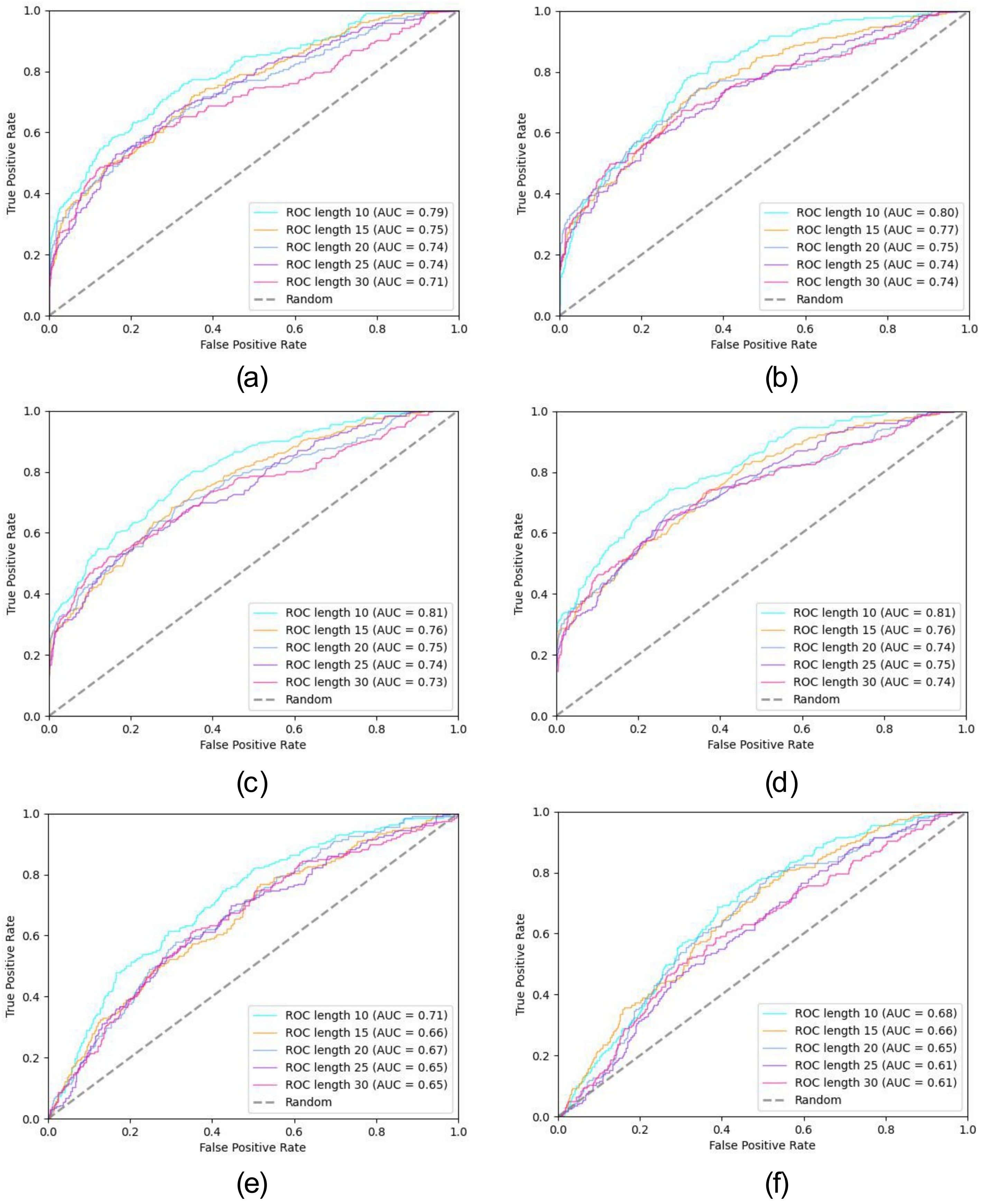

Figure s2: ROC curves of incorporated features. (a). incorporation of RRC and RRPC in SVM classifier, (b). incorporation of AAC and AAPC in SVM classifier, (c). incorporation of RRC and RRPC in RF classifier, (d). incorporation of AAC and AAPC in RFclassifier, (e). incorporation of RRC and RRPC in LR classifier, (f). incorporation of AAC and AAPC in LR classifier.

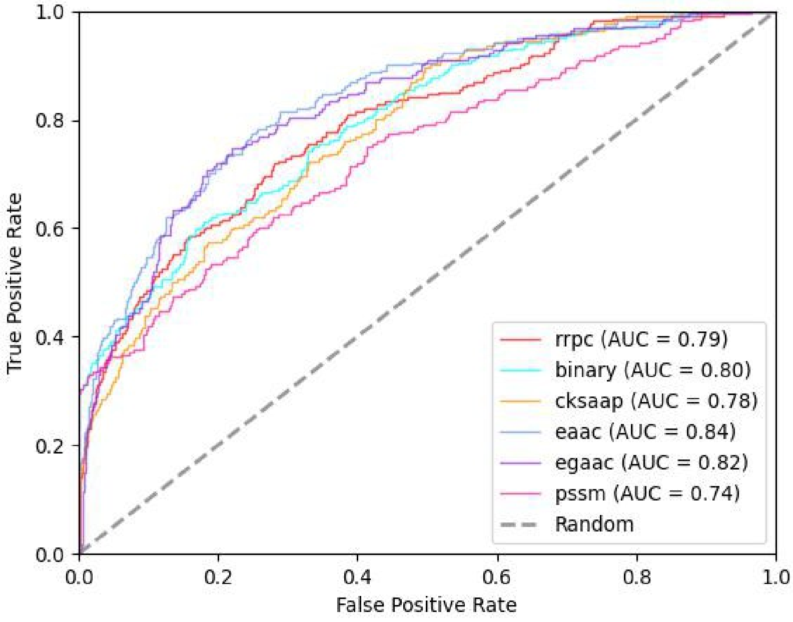

(a)

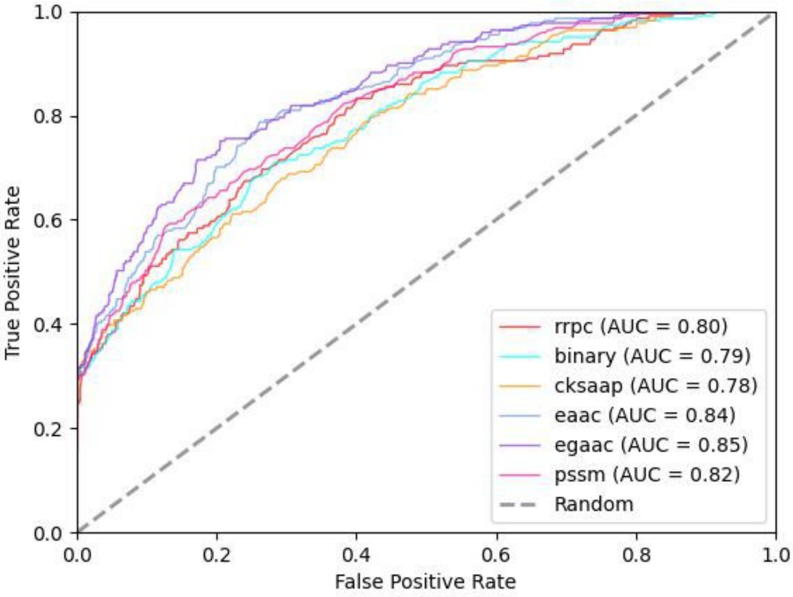

(b)

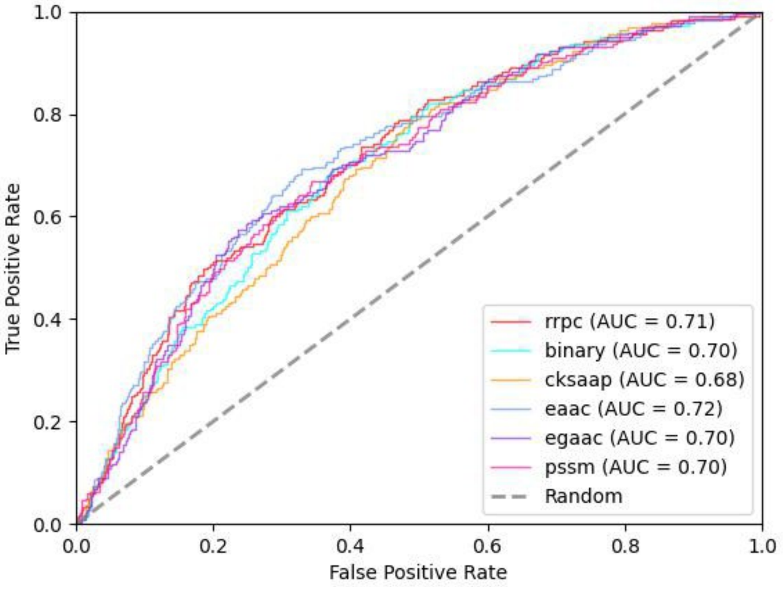

(c)

Figure s3: ROC curves of both sequence-based features and residue-residue contact based feature in (a). SVM classifier, (b). RF classifier, (c) LR classifier.

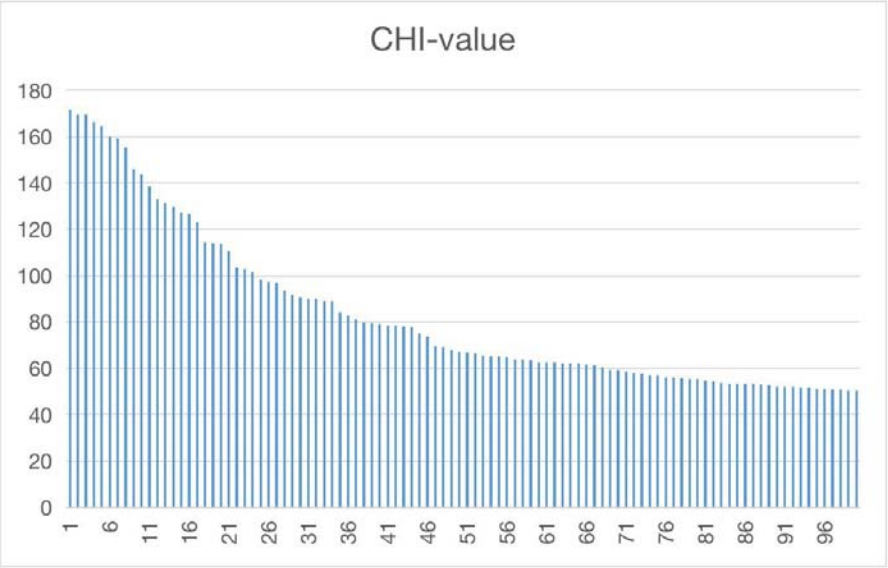

(a)

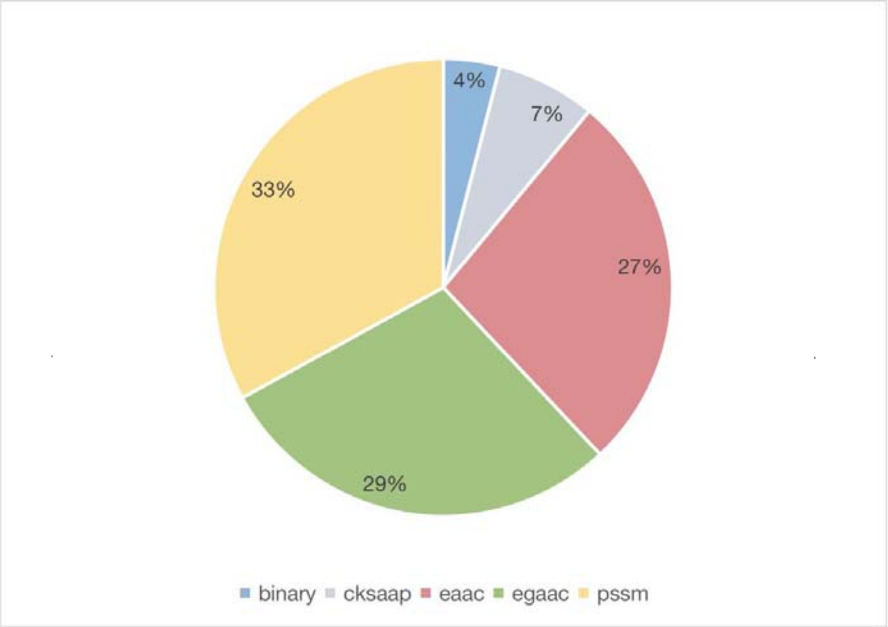

(b)

1	SW.10.negativecharger	21	Pos.10.Y	41	SW.12.postivecharger	61	SW.3.R	81	ER.gap3		
2	SW.4.postivecharger	22	Pos.10.D	42	SW.14.R	62	SW.4.R	82	KK.gap1	binary	4
3	SW.2.postivecharger	23	SW.2.K	43	SW.8.postivecharger	63	SW.9.D	83	SW.11.postivecharger	cksaap	7
4	SW.3.postivecharger	24	Pos.10.F	44	Pos.10.T	64	BINARY.F192	84	SW.8.K	eaac	27
5	Pos.10.K	25	SW.4.K	45	SW.9.postivecharger	65	Pos.2.E	85	SW.14.uncharger	egaac	29
6	SW.9.negativecharger	26	SW.3.K	46	SW.12.E	66	SW.13.negativecharger	86	RI.gap1	pssm	33
7	Pos.10.Q	27	Pos.12.E	47	SW.13.uncharger	67	SW.5.K	87	SW.11.K		
8	Pos.10.L	28	Pos.17.R	48	Pos.15.D	68	SW.13.R	88	SW.14.K		
9	Pos.10.R	29	SW.10.E	49	Pos.12.S	69	SW.17.R	89	Pos.18.K		
10	SW.14.postivecharger	30	SW.17.postivecharger	50	SW.8.E	70	Pos.5.K	90	SW.5.uncharger		
11	SW.15.postivecharger	31	Pos.10.W	51	Pos.12.Q	71	KD.gap2	91	SW.9.K		
12	SW.12.negativecharger	32	Pos.17.K	52	Pos.12.L	72	SW.17.uncharger	92	SW.2.R		
13	Pos.10.E	33	Pos.17.Q	53	Pos.5.Q	73	SW.11.D	93	SW.6.negativecharger		
14	SW.11.negativecharger	34	SW.1.K	54	SW.10.D	74	SW.12.uncharger	94	SW.16.uncharger		
15	SW.16.postivecharger	35	SW.16.R	55	Pos.12.K	75	PQ.gap2	95	BINARY.F187		
16	SW.5.postivecharger	36	Pos.15.E	56	Pos.5.R	76	SW.11.E	96	BINARY.F227		
17	SW.1.postivecharger	37	SW.8.D	57	Pos.12.P	77	SW.10.postivecharger	97	ER.gap2		
18	SW.8.negativecharger	38	Pos.10.H	58	Pos.10.I	78	SW.15.K	98	DE.gap3		
19	SW.13.postivecharger	39	SW.15.R	59	Pos.19.K	79	SW.15.uncharger	99	Pos.11.R		
20	Pos.12.D	40	SW.9.E	60	Pos.10.M	80	BINARY.F322	100	Pos.4.Q		

(c)

Figure s4: Feature selection of method Chi-square(CHI2) without any spatial-based feature involved. (a). The top-100 CHI2 value in descending order. (b). the percentage of each sequence-based feature in with the top-100 large CHI2 value. (c). the detail top-100 feature selection with the largest 100 CHI2 values.

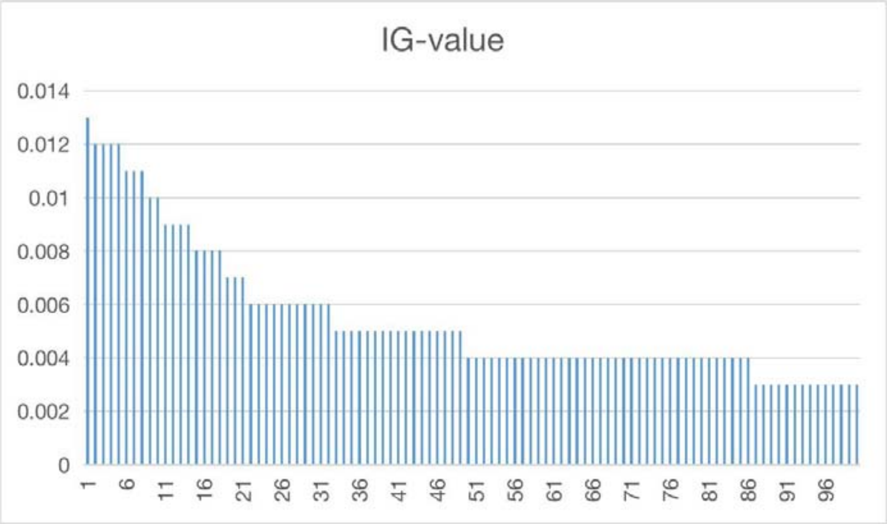

(a)

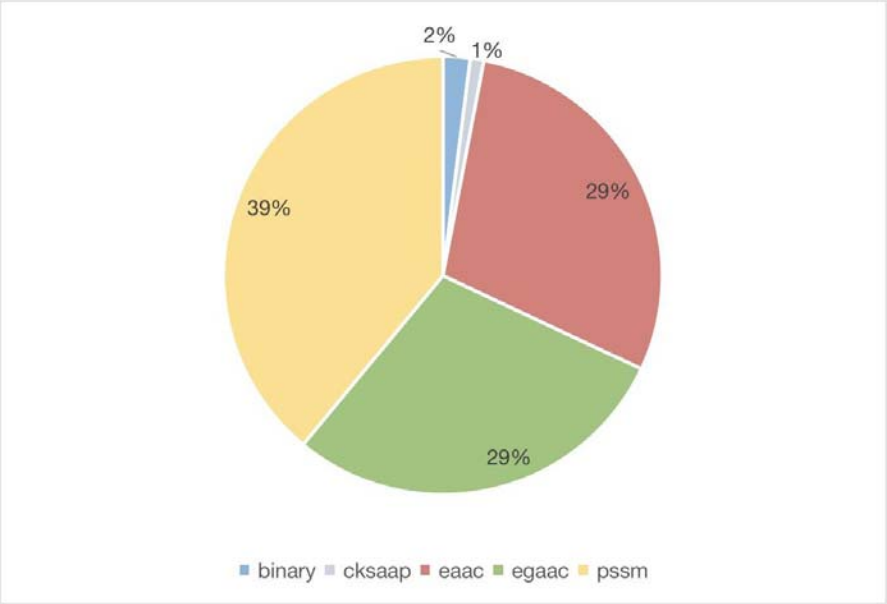

(b)

1	Pos.10.K	21	SW.8.postivecharger	41	SW.16.R	61	BINARY.F182	81	SW.16.uncharger		
2	Pos.10.R	22	SW.9.postivecharger	42	SW.14.R	62	SW.12.uncharger	82	SW.15.K		
3	Pos.10.Q	23	SW.2.K	43	Pos.10.F	63	Pos.10.I	83	KK.gap1		
4	SW.4.postivecharger	24	SW.4.K	44	SW.10.postivecharger	64	SW.8.E	84	Pos.14.I	binary	2
5	SW.2.postivecharger	25	SW.3.K	45	SW.12.E	65	SW.7.postivecharger	85	SW.11.E	cksaap	1
6	SW.3.postivecharger	26	SW.17.postivecharger	46	SW.9.K	66	SW.15.uncharger	86	Pos.5.K	eaac	29
7	SW.10.negativecharger	27	Pos.12.E	47	Pos.10.H	67	SW.5.K	87	SW.11.D	egaac	29
8	Pos.10.L	28	SW.10.E	48	SW.8.K	68	Pos.15.D	88	Pos.12.Y	pssm	39
9	SW.9.negativecharger	29	BINARY.F192	49	Pos.12.P	69	Pos.5.R	89	SW.8.D		
10	SW.14.postivecharger	30	Pos.10.D	50	Pos.12.L	70	SW.10.K	90	SW.14.K		
11	SW.15.postivecharger	31	Pos.10.Y	51	Pos.12.S	71	SW.4.R	91	Pos.14.T		
12	Pos.10.E	32	SW.1.K	52	SW.15.R	72	SW.3.R	92	Pos.10.V		
13	SW.16.postivecharger	33	Pos.17.R	53	Pos.12.K	73	SW.10.D	93	Pos.2.E		
14	SW.5.postivecharger	34	Pos.17.Q	54	Pos.10.M	74	Pos.11.R	94	SW.17.R		
15	SW.12.negativecharger	35	Pos.10.W	55	Pos.15.E	75	SW.14.uncharger	95	Pos.4.Q		
16	SW.1.postivecharger	36	SW.12.postivecharger	56	SW.17.uncharger	76	SW.13.R	96	SW.7.K		
17	SW.11.negativecharger	37	Pos.17.K	57	SW.13.negativecharger	77	SW.5.uncharger	97	SW.2.R		
18	SW.13.postivecharger	38	SW.13.uncharger	58	Pos.12.Q	78	Pos.11.K	98	Pos.17.F		
19	SW.8.negativecharger	39	SW.9.E	59	Pos.5.Q	79	SW.9.D	99	Pos.19.K		
20	Pos.12.D	40	Pos.10.T	60	SW.11.postivecharger	80	SW.11.K	100	Pos.16.R		

(c)

Figure s5: Feature selection of method information-gain(IG) without any spatial-based feature involved. (a). The top-100 IG value in descending order. (b). the percentage of each sequence-based feature in with the top-100 large IG value. (c). the detail top-100 feature selection with the largest 100 IG values.

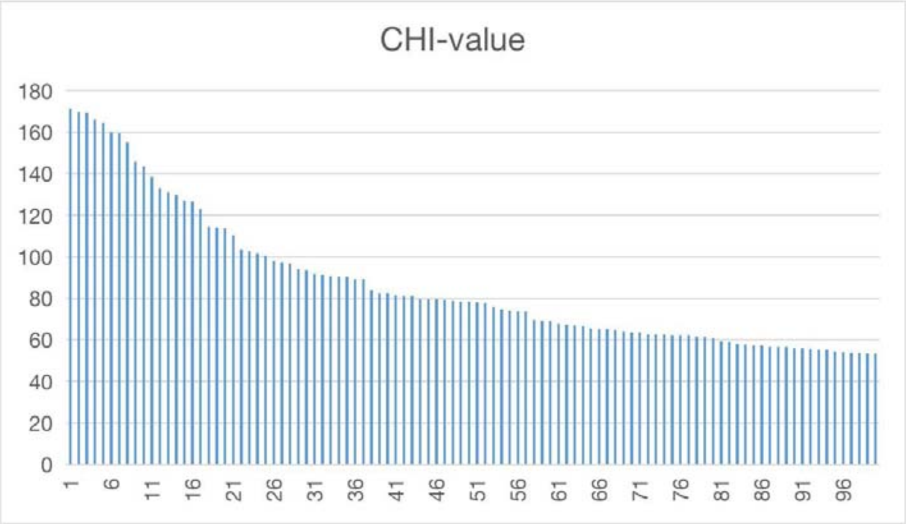

(a)

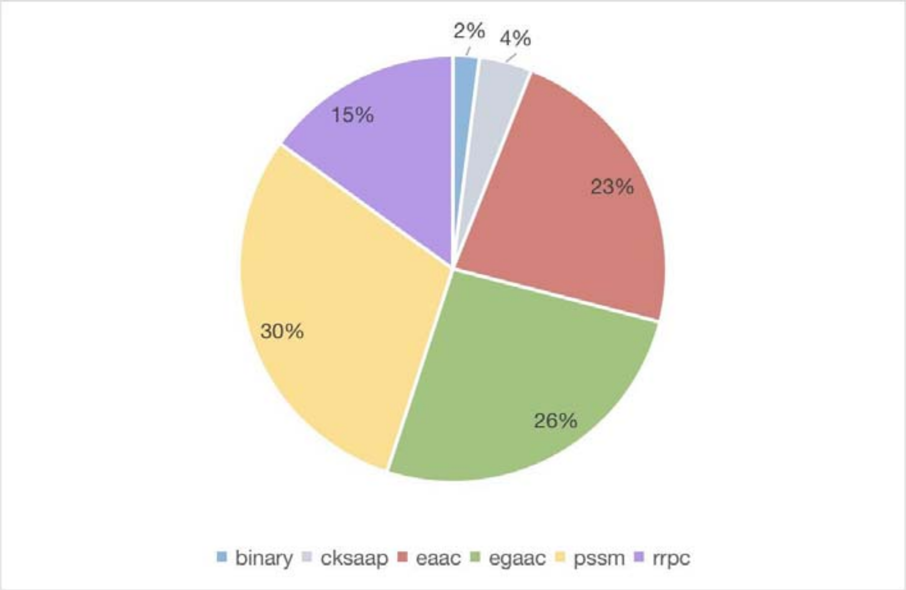

(b)

1	SW.10.negativecharger	21	Pos.10.Y	41	VK	61	Pos.12.S	81	SW.17.R		
2	SW.4.postivecharger	22	Pos.10.D	42	KI	62	SW.8.E	82	Pos.5.K		
3	SW.2.postivecharger	23	SW.2.K	43	SW.8.D	63	Pos.12.Q	83	IQ		
4	SW.3.postivecharger	24	Pos.10.F	44	Pos.10.H	64	Pos.12.L	84	SW.17.uncharger	binary	2
5	Pos.10.K	25	EK	45	MK	65	Pos.5.Q	85	SW.11.D	cksaap	4
6	SW.9.negativecharger	26	SW.4.K	46	SW.15.R	66	SW.10.D	86	KG	eaac	23
7	Pos.10.Q	27	SW.3.K	47	AK	67	Pos.12.K	87	SW.12.uncharger	egaac	26
8	Pos.10.L	28	Pos.12.E	48	SW.9.E	68	Pos.5.R	88	PQ.gap2	pssm	30
9	Pos.10.R	29	RI	49	SW.12.postivecharger	69	Pos.12.P	89	KD.gap2	rrpc	15
10	SW.14.postivecharger	30	Pos.17.R	50	SW.14.R	70	Pos.10.I	90	SW.11.E		
11	SW.15.postivecharger	31	EL	51	SW.8.postivecharger	71	Pos.19.K	91	SW.10.postivecharger		
12	SW.12.negativecharger	32	SW.10.E	52	Pos.10.T	72	Pos.10.M	92	SW.15.K		
13	Pos.10.E	33	SW.17.postivecharger	53	KD	73	SW.3.R	93	SW.15.uncharger		
14	SW.11.negativecharger	34	Pos.10.W	54	SW.9.postivecharger	74	SW.4.R	94	BINARY.F322		
15	SW.16.postivecharger	35	Pos.17.K	55	DG	75	SW.9.D	95	ER.gap3		
16	SW.5.postivecharger	36	Pos.17.Q	56	SW.12.E	76	BINARY.F192	96	KK.gap1		
17	SW.1.postivecharger	37	SW.1.K	57	KE	77	Pos.2.E	97	SW.11.postivecharger		
18	SW.8.negativecharger	38	SW.16.R	58	PQ	78	SW.13.negativecharger	98	EA		
19	SW.13.postivecharger	39	KA	59	SW.13.uncharger	79	SW.5.K	99	SW.8.K		
20	Pos.12.D	40	Pos.15.E	60	Pos.15.D	80	SW.13.R	100	SW.14.uncharger		

(c)

Figure s6: Feature selection of method Chi-square(CHI2) with spatial-based feature RRPC involved. (a). The top-100 CHI2 value in descending order. (b). the percentage of each sequence-based feature in with the top-100 large CHI2 value. (c). the detail top-100 feature selection with the largest 100 CHI2 values. The reason for choosing RRPC is due to its larger diversity in the positive and negative samples than RRC feature.

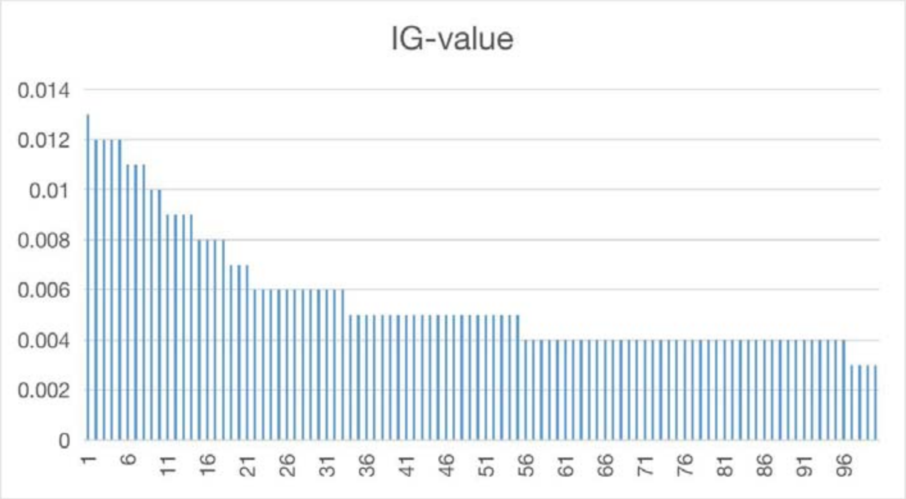

(a)

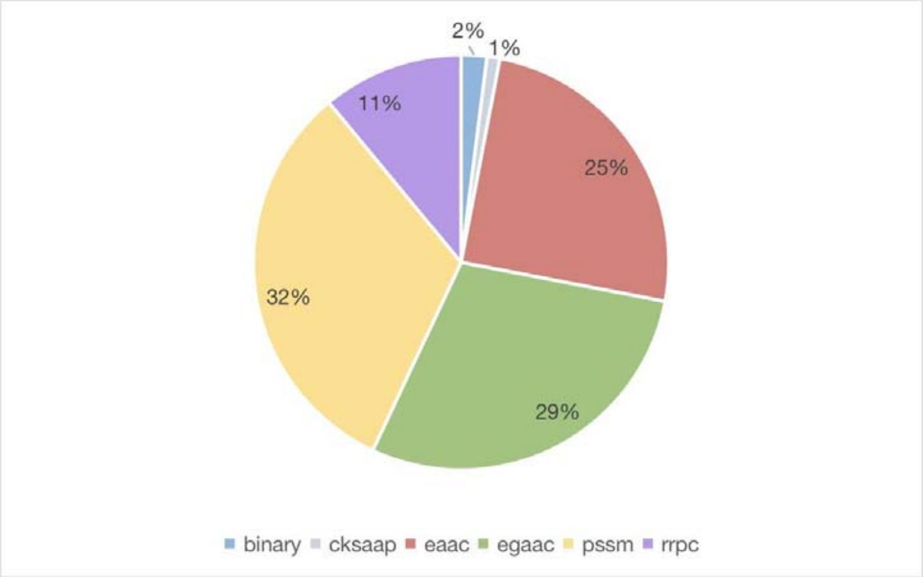

(b)

1	Pos.10.K	21	SW.8.postivecharger	41	Pos.17.K	61	Pos.15.E	81	SW.3.R		
2	Pos.10.R	22	SW.9.postivecharger	42	SW.13.uncharger	62	SW.17.uncharger	82	SW.10.D		
3	Pos.10.Q	23	SW.2.K	43	SW.9.E	63	SW.13.negativecharger	83	Pos.11.R	binary	2
4	SW.4.postivecharger	24	SW.4.K	44	Pos.10.T	64	Pos.12.Q	84	SW.14.uncharger	cksaap	1
5	SW.2.postivecharger	25	SW.3.K	45	AK	65	Pos.5.Q	85	SW.13.R	eaac	25
6	SW.3.postivecharger	26	SW.17.postivecharger	46	SW.16.R	66	SW.11.postivecharger	86	SW.5.uncharger	egaac	29
7	SW.10.negativecharger	27	Pos.12.E	47	SW.14.R	67	BINARY.F182	87	Pos.11.K	pssm	32
8	Pos.10.L	28	SW.10.E	48	Pos.10.F	68	SW.12.uncharger	88	SW.9.D	rrpc	11
9	SW.9.negativecharger	29	BINARY.F192	49	SW.10.postivecharger	69	VK	89	SW.11.K		
10	SW.14.postivecharger	30	EK	50	SW.12.E	70	Pos.10.I	90	KG		
11	SW.15.postivecharger	31	Pos.10.D	51	SW.9.K	71	KE	91	SW.16.uncharger		
12	Pos.10.E	32	Pos.10.Y	52	Pos.10.H	72	SW.8.E	92	SW.15.K		
13	SW.16.postivecharger	33	SW.1.K	53	SW.8.K	73	EL	93	KK.gap1		
14	SW.5.postivecharger	34	Pos.17.R	54	KD	74	SW.7.postivecharger	94	Pos.14.I		
15	SW.12.negativecharger	35	RI	55	Pos.12.P	75	SW.15.uncharger	95	SW.11.E		
16	SW.1.postivecharger	36	Pos.17.Q	56	Pos.12.L	76	SW.5.K	96	Pos.5.K		
17	SW.11.negativecharger	37	KI	57	Pos.12.S	77	Pos.15.D	97	SW.11.D		
18	SW.13.postivecharger	38	Pos.10.W	58	SW.15.R	78	Pos.5.R	98	Pos.12.Y		
19	SW.8.negativecharger	39	KA	59	Pos.12.K	79	SW.10.K	99	MK		
20	Pos.12.D	40	SW.12.postivecharger	60	Pos.10.M	80	SW.4.R	100	SW.8.D		

(c)

Figure s7: Feature selection of method information-gain(IG) with spatial-based feature RRPC involved. (a). The top-100 IG value in descending order. (b). the percentage of each sequence-based feature in with the top-100 large IG value. (c). the detail top-100 feature selection with the largest 100 IG values.

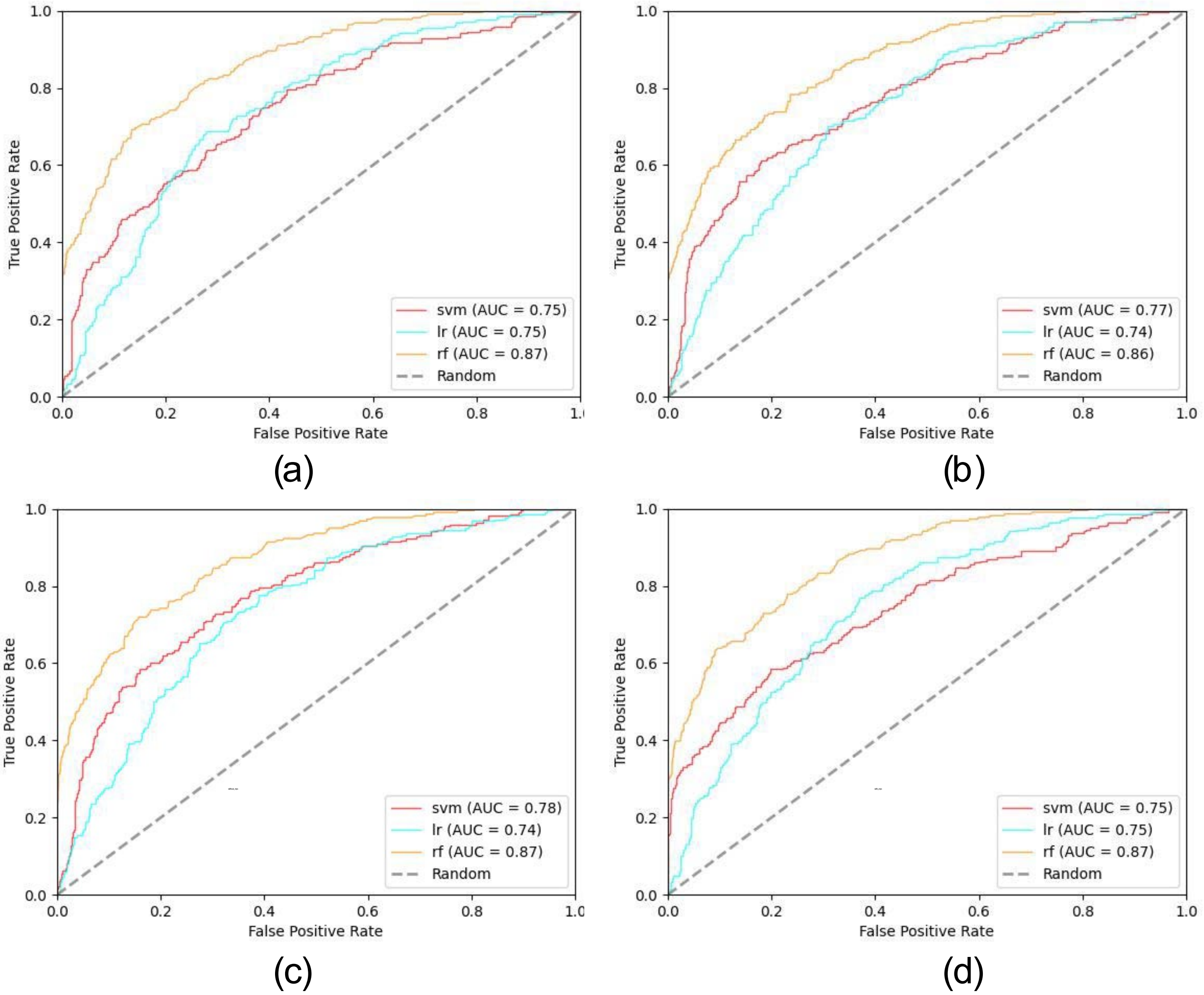

Figure s8: ROC curves of feature selection methods Chi-square(Chi2) and information-gain(IG) in the cases of with or without RRPC incorporated. (a). Chi2 method without RRPC incorporated. (b). Chi2 method with RRPC incorporated. (c). IG method without RRPC incorporated. (d). IG method with RRPC incorporated.
